# The Chemical Composition of *Achillea wilhelmsii* C. Koch and Its Desirable Effects on Hyperglycemia, Inflammatory Mediators and Hypercholesterolemia as Risk Factors for Cardiometabolic Disease

**DOI:** 10.3390/molecules21040404

**Published:** 2016-03-25

**Authors:** Elian Khazneh, Petra Hřibová, Jan Hošek, Pavel Suchý, Peter Kollár, Gabriela Pražanová, Jan Muselík, Zuzana Hanaková, Jiří Václavík, Michał Miłek, Jaroslav Legáth, Karel Šmejkal

**Affiliations:** 1 Department of Natural Drugs, Faculty of Pharmacy, University of Veterinary and Pharmaceutical Sciences, Palackého tř. 1, Brno 61242, Czech Republic; cerfpatolog@gmail.com (P.H.); hanakovaz@vfu.cz (Z.H.); vaclavikj@vfu.cz (J.V.); karel.mejkal@post.cz (K.Š.); 2 Department of Molecular Biology and Pharmaceutical Biotechnology, Faculty of Pharmacy, University of Veterinary and Pharmaceutical Sciences, Palackého tř. 1, Brno 61242, Czech Republic; hhosek@gmail.com; 3 Department of Human Pharmacology and Toxicology, Faculty of Pharmacy, University of Veterinary and Pharmaceutical Sciences, Palackého tř. 1, Brno 61242, Czech Republic; suchypa@vfu.cz (P.S.); kollarp@vfu.cz (P.K.); prazanovag@vfu.cz (G.P.); 4 Department of Pharmaceutics, Faculty of Pharmacy, University of Veterinary and Pharmaceutical Sciences, Palackého 1-3, Brno 61242, Czech Republic; muselikj@vfu.cz; 5 Department of Biotechnology and Bioinformatics, Faculty of Chemistry, Rzeszów University of Technology, Powstańców Warszawy 6, Rzeszów 35-959, Poland; mkkmilek@gmail.com (M.M.); Jaroslav.Legath@uvlf.sk (J.L.); 6 Department of Pharmacology and Toxicology, The University of Veterinary Medicine and Pharmacy in Košice, Komenského 73, Košice 04181, Slovakia

**Keywords:** *Achillea wilhelmsii*, anti-hypercholesterolemic, cardiometabolic disease, docking, flavonoids, 3-hydroxy-3-methylglutaryl-CoA reductase, hypoglycemic, inflammation

## Abstract

This study was done to identify the content compounds of *Achillea wilhelmsii* (*A. wilhelmsii*) and to evaluate its hypoglycemic and anti-hypercholesterolemic activity and effect on inflammatory mediators. The extracts and fractions of *A. wilhelmsii* were thoroughly analyzed using high performance liquid chromatography (HPLC), and the total content of phenols and flavonoids was determined. The hypoglycemic activity was evaluated *in vivo* using alloxan-induced diabetic mice. The effect upon inflammatory mediators was evaluated *in vitro* using the human monocytic leukemia cell line (THP-1). The anti-hypercholesterolemic activity was evaluated *in vitro* using the 3-hydroxy-3-methylglutaryl-CoA (HMG-CoA) reductase assay kit. The water extract (WE)-treated group showed the highest reduction in the fasting blood glucose levels (FBGL). The chloroform fraction (CF) and ethyl acetate fraction (EAF) both showed a significant ability to reduce the secretion of tumor necrosis factor alpha (TNF-α). The EAF, however, also attenuated the levels of matrix metalloproteinase-2 (MMP-2) and matrix metalloproteinase-9 (MMP-9). The CF showed the most significant 3-hydroxy-3-methylglutaryl-CoA reductase (HMGR) inhibition activity. The five main compounds in the CF were isolated and identified. Out of the five compounds in the CF, 1β,10β-epoxydesacetoxymatricarin (CP1) and leucodin (CP2) showed the highest anti-hypercholesterolemic potential. A molecular docking study provided corresponding results.

## 1. Introduction

*Achillea* is an herb that belongs to the family Asteraceae. This genus includes more than 100 species worldwide [1]. *Achillea* species occur in the high mountains of the Mediterranean [2] and are native to Europe and Western Asia [1]. Due to their medicinally-useful properties, several *Achillea* species have been used since ancient times as traditional remedies for abdominal pain, cough, inflammation, jaundice, fever, diarrhea and wound healing [3,4]. Various species of *Achillea* have been analyzed, and more than 100 compounds have been identified [1]. *Achillea* extracts showed anti-oxidant, anti-inflammatory, analgesic, anti-pyretic, anti-spasmodic, anti-ulcerogenic, anti-bacterial, cytotoxic, immunosuppressive and hypoglycemic properties [2,3,4,5,6,7,8,9,10,11]. *Achillea wilhelmsii* (*A. wilhelmsii*) C. Koch has previously demonstrated anti-hyperlipidemic, anti-hypertensive and anti-mycobacterial properties [12,13].

Diabetes mellitus (DM) is a metabolic disorder that is primarily characterized by hyperglycemia. This disease had been known and treated with traditional herbal therapies long before the chemical agents we know today became available [14].

Hypercholesterolemia, a form of hyperlipidemia, is nowadays, together with other non-communicable diseases, such as myocardial infarction, stroke, obesity and diabetes, among the leading causes of death worldwide in all income groups equally [15]. It is the combination of genetic and environmental risk factors that is responsible for the high levels of cholesterol in blood [16]. Nowadays, one of the most effective ways of lowering plasma cholesterol levels is controlling *de novo* synthesis of endogenous cholesterol by the inhibition of HMGR. The inhibition of this enzyme represents the rate-limiting step of the mevalonate pathway for the synthesis of sterol isoprenoids, such as cholesterol, and non-sterol isoprenoids, such as dolichol, heme-A, isopentenyl tRNA and ubiquinone [17,18].

A leading cause of premature death is cardiovascular disease, arising from increased rates of cardiometabolic risk factors, such as obesity, hypertension, hyperglycemia and dyslipidemia [19]. Inflammation is a key component of several cardiometabolic diseases, including obesity, type II diabetes and atherosclerotic cardiovascular disease [20]. Cardiometabolic risk factors, including elevated lipids, glucose and hypertension, are expected to continue to rise, especially as the population ages [21]. Adipose tissue in abdominal obesity is considered as an endocrinal organ orchestrating key pathophysiological pathways in inflammation and lipid metabolism. Adipose tissue synthesizes and secretes various adipocytokines that create a pro-inflammatory environment [22,23,24].

This study was done to evaluate the hypoglycemic and anti-hypercholesterolemic activities of *A. wilhelmsii* and to explore its influence on selected inflammatory mediators. To the best of our knowledge, this is the first time this plant has been studied thoroughly for its desirable effects on the major risk factors for cardiometabolic disease.

## 2. Results

### 2.1. Phytochemical Analyses

**Figure 1:**
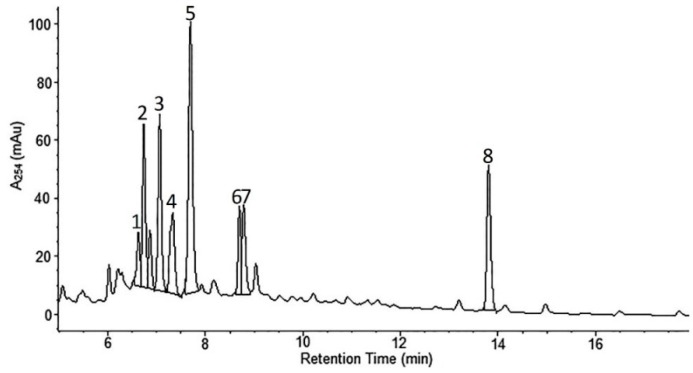
The selected segment of HPLC-DAD chromatogram of the water extract (WE) recorded at 254 nm. Peak assignments (tentative identification): 1. isoschaftoside; 2. schaftoside; 3. vicenin-2; 4. vicenin-3; 5. caffeic acid; 6. isoorientin; 7. isovitexin; 8. leucodin.

**Figure 2:**
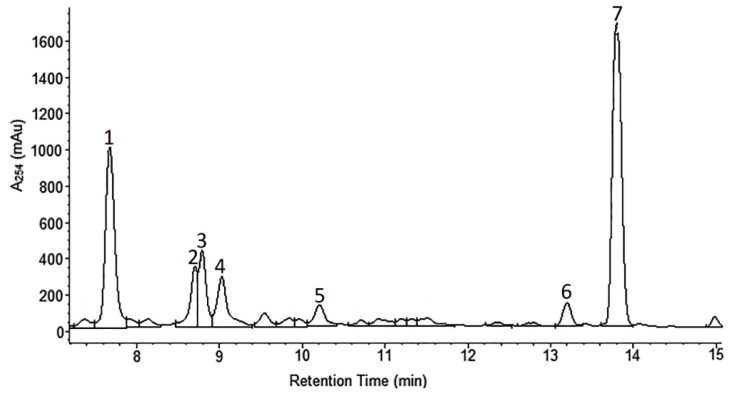
The selected segment of HPLC-DAD chromatogram of the ethyl acetate fraction (EAF) recorded at 254 nm. Peak assignments (tentative identification): 1. caffeic acid; 2. isoorientin; 3. isovitexin; 4. swertisin; 5. ferulic acid; 6. 1β,10β-epoxydesacetoxymatricarin; 7. leucodin.

**Figure 3:**
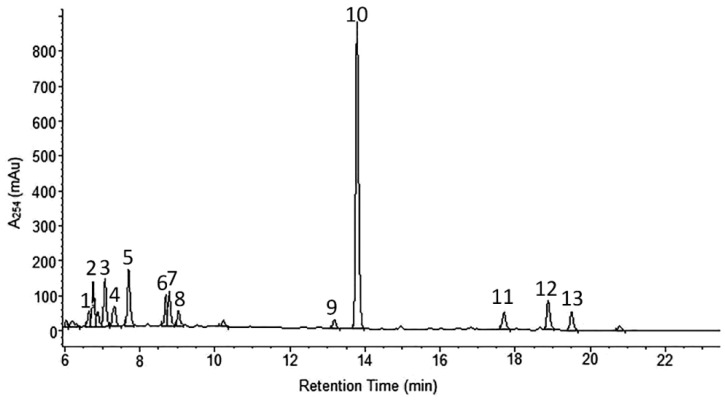
The selected segment of HPLC-DAD chromatogram of the hydro-alcoholic extract (HAE) recorded at 254 nm. Peak assignments (tentative identification): 1. isoschaftoside; 2. schaftoside; 3. vicenin-2; 4. vicenin-3; 5. caffeic acid; 6. isoorientin; 7. isovitexin; 8. swertisin; 9. 1β,10β-epoxydesacetoxymatricarin; 10. leucodin; 11. 5-demethylsinensetin; 12. 2-(3,4-dimethoxy-phenyl)-3-hydroxy-5,6,7-trimethoxy-chromen-4-one; 13. salvigenin.

**Figure 4:**
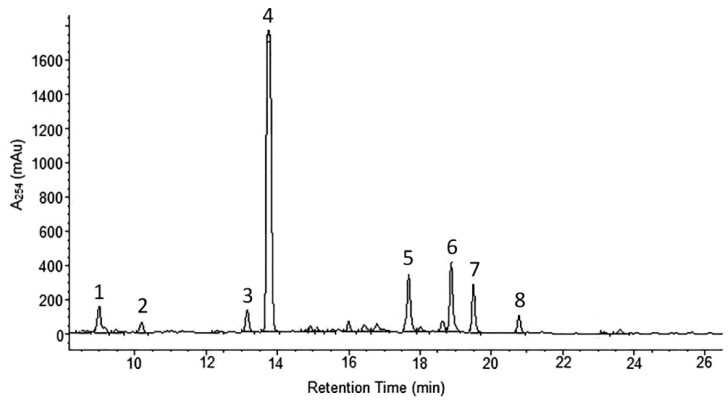
The selected segment of HPLC-DAD chromatogram of the chloroform fraction (CF) recorded at 254 nm. Peak assignments (tentative identification): 1. unknown; 2. ferulic acid; 3. 1β,10β-epoxydesacetoxymatricarin (CP1); 4. leucodin (CP2); 5. 5-demethylsinensetin (CP3); 6. 2-(3,4-dimethoxy-phenyl)-3-hydroxy-5,6,7-trimethoxy-chromen-4-one (CP4); 7. salvigenin (CP5); 8. unknown.

**Figure 5:**
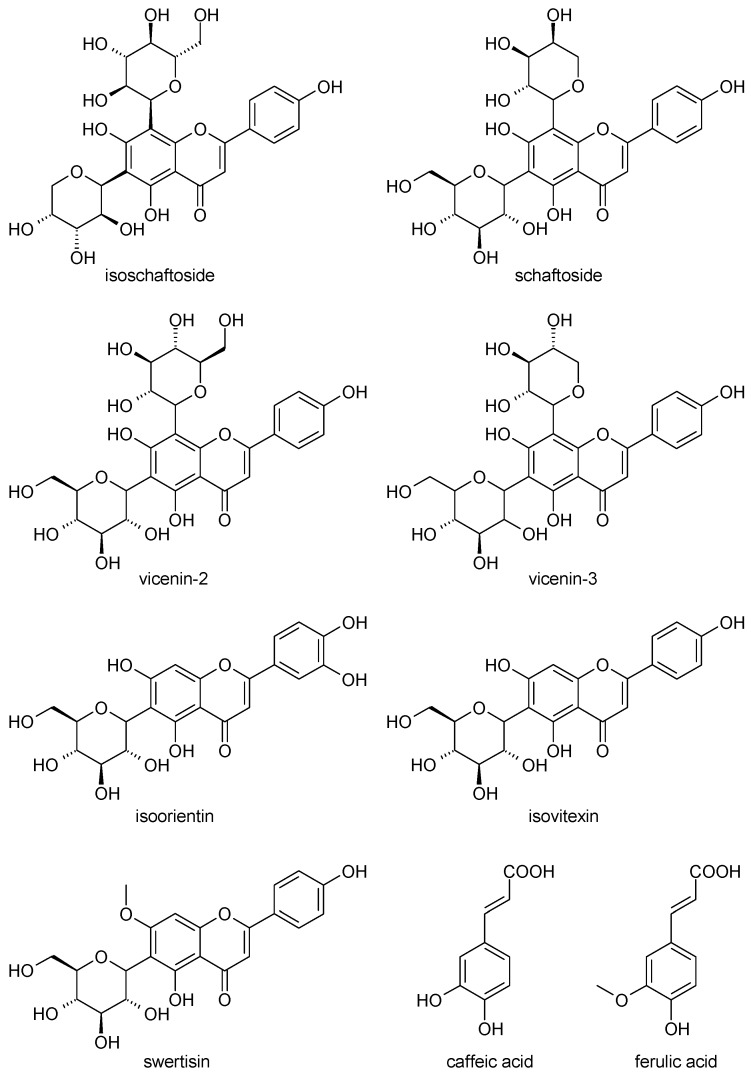
The structures of the compounds identified tentatively in *A. wilhelmsii.*

**Figure 6:**
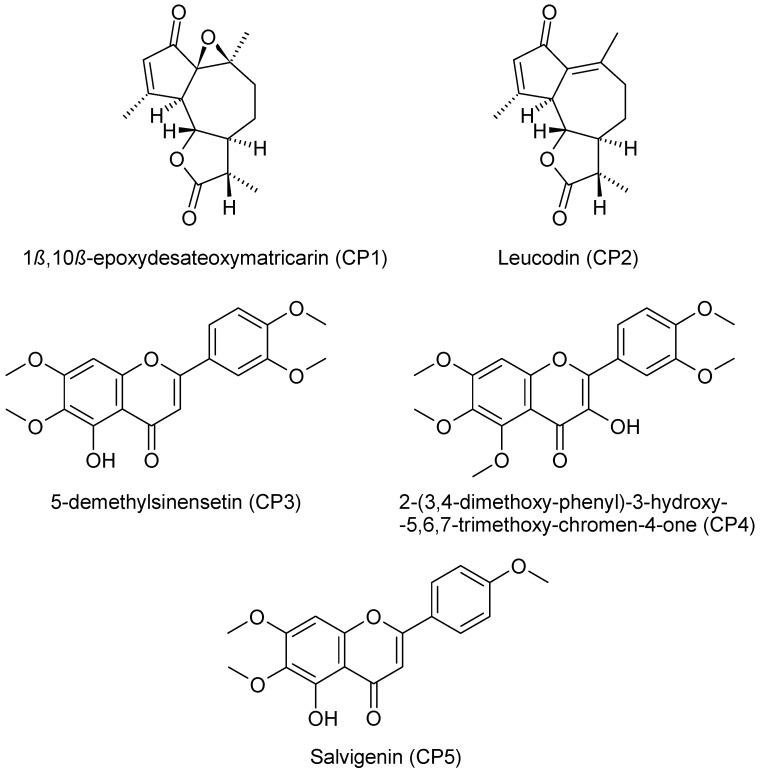
The structures of the compounds isolated and identified from the CF of *A. wilhelmsii.*

*A. wilhelmsii* extracts and fractions, the water extract (WE), the ethyl acetate fraction (EAF), the hydro-alcoholic extract (HAE) and the chloroform fraction (CF), were examined using high performance liquid chromatography (HPLC) with the aim to analyze their content of phenolic compounds. The compounds present were identified by their UV spectra and mass spectrometric ions through library search and comparison with the literature. It is important to note that this identification is tentative. The profile of the different phenolic compounds in the WE is shown in Figure 1. *C*-glycosides of luteolin and apigenin are the most abundant components. Caffeic acid is also present. The EAF chromatogram is shown in Figure 2. *C*-glycosides, ferulic acid and two sesquiterpenoids, 1β,10β-epoxydesacetoxymatricarin (CP1) and leucodin (CP2), are present. The same two sesquiterpenoid compounds are also present in the HAE and CF. Three methoxylated flavonoid aglycones are present in the HAE and CF: 2-(3,4-dimethoxyphenyl)-5-hydroxy-6,7-dimethoxychromen-4-one (CP3), 2-(3,4-dimethoxyphenyl)-5,6,7-dimethoxychromen-4-one (CP4) and salvigenin (CP5). Figure 3 and Figure 4 show the chromatograms of the HAE and CF, respectively. Figure 5 shows the structures of the compounds identified tentatively in *A. wilhelmsii*. Figure 6 shows the structures of the compounds isolated and identified from the CF of *A. wilhelmsii*.

The total content of polyphenols was determined by a modified Folin-Ciocalteu colorimetric method, and the results were expressed as gallic acid equivalent (GAE) per gram of dry extract weight. Flavone and flavonol contents were analyzed using a colorimetric method, and the results were expressed as quercetin equivalent (QE) per gram of dry extract weight [25]. The findings are summarized in Table 1. EAF showed the highest content of phenols among the tested samples, while CF showed the highest content of flavonoids. The WE showed the least content of phenols and flavonoids.

### 2.2. The Hypoglycemic Activity

The hypoglycemic activity was tested *in vivo.* Plant extracts and fractions were administered for 20 consecutive days. Quercetin was used as a standard for comparison. The results of the hypoglycemic activity assay are summarized in Table 2. On the first day of the experiment, the differences in the fasting blood glucose levels (FBGL) of the diabetic groups were statistically insignificant when compared to each other. On the fourth day, the FBGL of both EAF- and quercetin-treated groups started to show a significant difference when compared to the diabetic group. On the last day of the experiment, the WE-, EAF- and HAE-treated groups showed significant differences when compared to the diabetic groups. However, the differences of the FBGL of the WE-treated group were statistically insignificant when compared to the healthy group.

The histology study revealed signs of acute enteritis in the jejunum of the CF-treated group that were not present in the other groups. The control diabetic group exhibited severe liver tissue injuries and lipid accumulations, shrinkage and severe damage of Langerhans islets with fat deposits and tubular edema and glomerular hemorrhage in the juxtamedullary region of the kidney. All of these morphological changes can be attributed to the administration of alloxan [26,27]. Both the WE- and quercetin-treated groups presented a higher number of functional Langerhans islets when compared to the other treated groups. Improvements in the liver and kidney tissue were also observed in the WE- and quercetin-treated groups.

### 2.3. The Effect on Inflammatory Mediators

The effect on inflammatory mediators of *A. wilhelmsii* was evaluated *in vitro* using the model of LPS-stimulated macrophage-like cells THP-1. The production of pro-inflammatory cytokine tumor necrosis factor alpha (TNF-α), and the activity of inflammation-related proteinases, matrix metalloproteinase-2 (MMP-2) and matrix metalloproteinase-9 (MMP-9) were used as markers of the inflammatory response in these cells.

**Figure 7:**
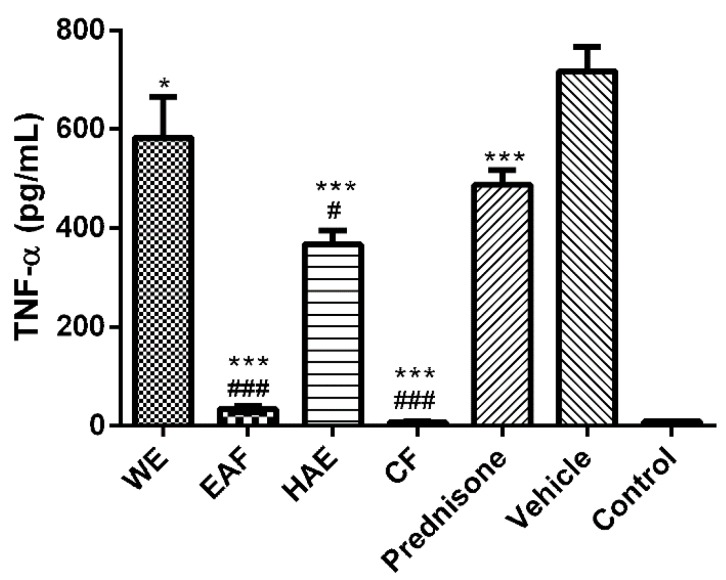
Effects of the tested *A. wilhelmsii* extracts and fractions and the reference drug prednisone, on TNF-α secretion. Cells were pre-treated with the given extracts and fractions (25 μg/mL), prednisone (1 μM) or the vehicle (DMSO) only. After 1 h of incubation, the inflammatory response was induced by the addition of lipopolysaccharide (LPS) (except for the control cells). Results are expressed as means ± SD for three independent experiments. * Significant difference in comparison to vehicle-treated cells (*p* < 0.05); *** significant difference in comparison to vehicle-treated cells (*p* < 0.001); # significant difference in comparison to prednisone-treated cells (*p* < 0.05); ^###^ significant difference in comparison to prednisone-treated cells (*p* < 0.001).

**Figure 8:**
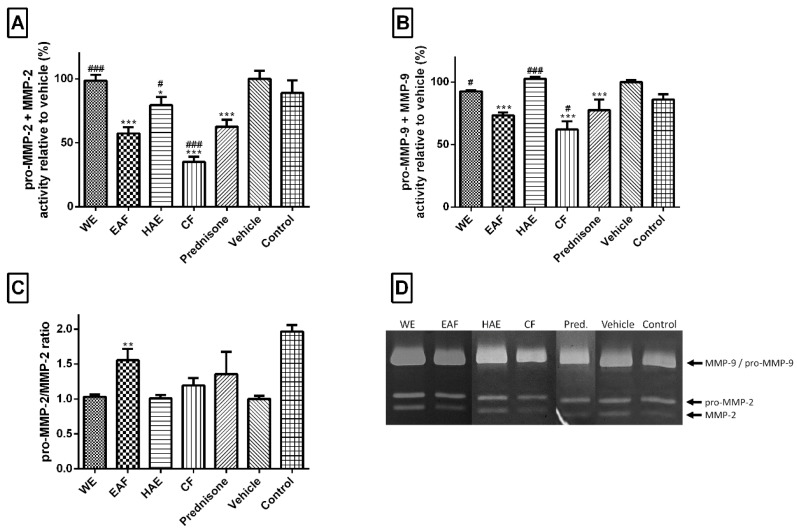
Effects of tested *A. wilhelmsii* extracts and fractions and the reference drug prednisone on LPS‑induced matrix metalloproteinases (MMP) activity. Cells were pre-treated with given extracts (25 μg/mL), prednisone (1 μM; Pred.) or the vehicle (DMSO) only. After 1 h of incubation, the inflammatory response was induced by the addition of LPS (except for the control cells). The activities of MMP-2 (**A**) and MMP-9 (**B**) were detected by zymography. The intensity of the digested bands was analyzed by densitometry analysis; (**C**) The pro-MMP-2/MMP-2 ratio. The results are expressed as means ± SD for three independent experiments. * Significant difference in comparison to vehicle-treated cells (*p* < 0.05); ** significant difference in comparison to vehicle-treated cells (*p* < 0.01); *** significant difference in comparison to the vehicle-treated cells (*p* < 0.001); ^#^ significant difference in comparison to prednisone-treated cells (*p* < 0.05); ^###^ significant difference in comparison to prednisone-treated cells (*p* < 0.001); (**D**) The gel representative results of one of the three independent experiments.

All extracts tested significantly attenuated TNF-α secretion (Figure 7). EAF and CF reduced the level of this cytokine almost to the basal level (control cells). Three *A. wilhelmsii* extract fractions, EAF, HAE and CF, were significantly better than 1 µM prednisone, whereas the WE was not as effective as this commercially available drug. The next evaluated marker of inflammation was proteinase MMP-2 (Figure 8A). The EAF, HAE and CF all had the ability to reduce the amount of this enzyme. CF had an even greater effect than prednisone. For the biological activity of MMP-2, it is necessary to truncate the inactive pro-MMP-2 form to an active MMP-2 form. Thus, the ratio between pro-MMP-2 and MMP-2 is more important than the total amount of MMP-2 itself. From this point of view, only the EAF significantly changed the pro-MMP-2/MMP-2 ratio towards pro-MMP-2 (Figure 8C). A typical pro-inflammatory marker is an elevated level of MMP-9. Both the EAF and CF were able to significantly attenuate the level of this enzyme (Figure 8B).

### 2.4. The Anti-Hypercholesterolemic Activity

#### 2.4.1. The 3-Hydroxy-3-methyl-glutaryl-CoA Reductase Inhibition Assay

The results of the HMGR inhibition assays of the extract and fractions are summarized in Table 3.

**Figure 9:**
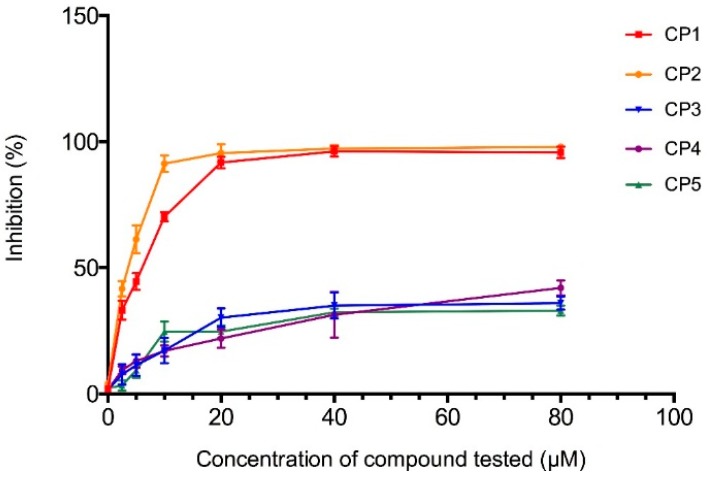
Inhibition (%) of each one of the five compounds (1β,10β-epoxydesacetoxymatricarin (CP1), leucodin (CP2), 5-demethylsinensetin (CP3), 2-(3,4-dimethoxy-phenyl)-3-hydroxy-5,6,7-trimethoxy-chromen-4-one (CP4) and salvigenin (CP5)) plotted against the tested concentrations (0, 2.5, 5, 10, 20, 40, 80 μM).

The CF showed the highest HMGR inhibition potential, so the five main compounds present in the CF (1β,10β-epoxydesacetoxymatricarin (CP1), leucodin (CP2), 5-demethylsinensetin (CP3), 2-(3,4-dimethoxy-phenyl)-3-hydroxy-5,6,7-trimethoxy-chromen-4-one (CP4) and salvigenin (CP5)) were isolated, identified (structures and spectral data are in the Supplementary Materials) and tested separately. The dose-dependent inhibition of HMGR for the compounds is shown in Figure 9. Both 1β,10β-epoxydesacetoxymatricarin (CP1) and leucodin (CP2) showed powerful inhibition of HMGR, whereas 5-demethylsinensetin (CP3), 2-(3,4-dimethoxy-phenyl)-3-hydroxy-5,6,7-trimethoxy-chromen-4-one (CP4) and salvigenin (CP5) did not reach 50% inhibition, even at higher concentrations (80 μM). The IC_50_ values for 1β,10β-epoxydesacetoxymatricarin (CP1) and leucodin (CP2) were 6.37 and 3.88 μM, respectively. For the standardization of the method, preliminary screening of pravastatin inhibition activity was performed. The IC_50_ value for pravastatin was 72.12 nM.

#### 2.4.2. Computational Docking

**Figure 10:**
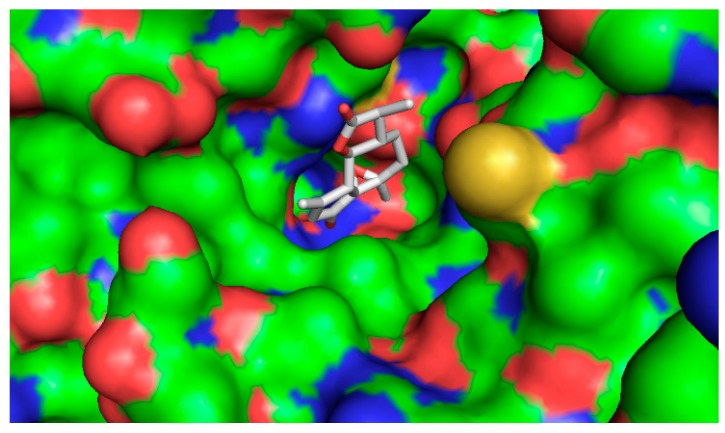
Bound conformer of ligands interacting with the substrate binding sites of HMGR for 1β,10β-epoxydesacetoxymatricarin (CP1).

**Figure 11:**
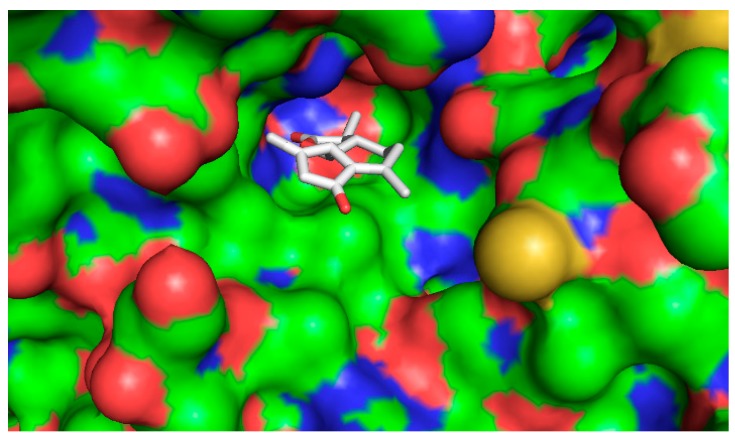
Bound conformer of ligands interacting with the substrate binding sites of HMGR for Leucodin (CP2).

**Figure 12:**
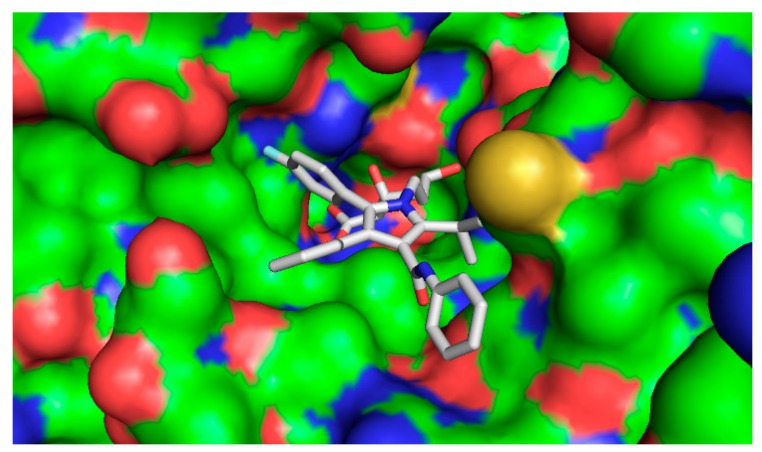
Bound conformer of ligands interacting with the substrate binding sites of HMGR (atorvastatin).

**Figure 13:**
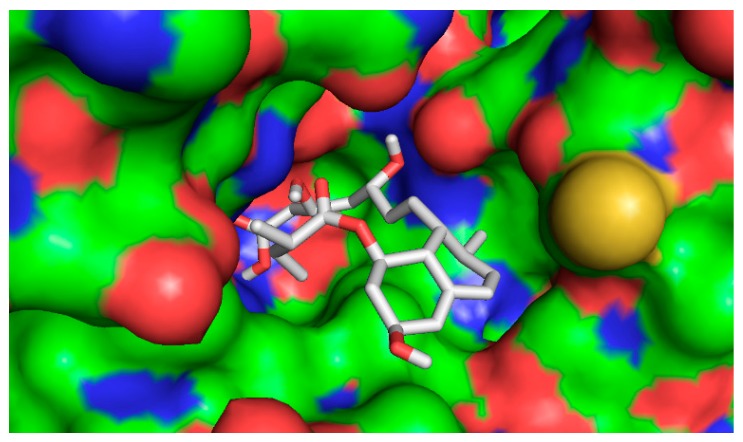
Bound conformer of ligands interacting with the substrate binding sites of HMGR (pravastatin).

The five compounds isolated from the CF were subjected to molecular docking study. For comparison purposes, statins were also docked into the active pocket to calculate the binding affinities to be compared with the active compounds found in CF. Statins had binding affinities that ranged from −7.0 to −9.6 Kcal/mol. Compounds 1β,10β-epoxydesacetoxymatricarin (CP1) and leucodin (CP2) (Figure 10 and Figure 11) showed the highest affinities and were perfectly docked into the active pocket site. Atorvastatin showed the highest affinity at −9.6 kcal/mol (Figure 12), while pravastatin had a binding affinity of −7.0 Kcal/mol (Figure 13). Similar findings were observed with the tested compounds with a range from −7.4 to −8.0 Kcal/mol (well within the range of the statins) (Table 4.). The slightly lower affinity in comparison with atorvastatin can be explained by the fact that 1β,10β-epoxydesacetoxymatricarin (CP1) and leucodin (CP2) are smaller molecules than atorvastatin with a smaller molecular surface and less specific shape for the binding site. It has to be noted that the other compounds, 5-demethylsinensetin (CP3), 2-(3,4-dimethoxy-phenyl)-3-hydroxy-5,6,7-trimethoxy-chromen-4-one (CP4) and salvigenin (CP5), also showed good binding affinity, but all the hits found by the docking program were not in the active pocket site, which corresponds with the fact that these compounds could not reach 50% inhibition at the tested concentrations.

## 3. Discussion

*C*-glycosides, especially luteolin and apigenin *C*-glycosides, were previously reported to occur in the genus *Achillea* [11,28]. Vicenin-2, schaftoside, isoschaftoside and isovitexin have all been reported from *Achillea setacea* [29]. Leucodin and deacetylmatricarin have been reported from *Achillea millefolium* [30]. Salvigenin has been reported before from *Achillea tenuifolia* [31]. Derivatives of caffeic acid and ferulic acid are also known to occur in the genus *Achillea* [32]. However, this is the first time the content compounds of *A. wilhelmsii* have been analyzed in detail. Our investigation led to the identification of two sesquiterpenoids and three methoxylated flavonoids that have not yet been reported in *A. wilhelmsii*. The determination of the total content of phenols and flavonoids enabled us to quantify the amount of the phenolic compounds present in *A. wilhelmsii*.

Quercetin is one of the most abundant flavonol-type flavonoids found in fruit and vegetables, and it is known to be a strong antioxidant. Several investigators reported the hypoglycemic effect of quercetin along with other important biological effects [14,33]. This was the reason why we used quercetin as a standard for comparison in the hypoglycemic activity assay. The results obtained showed that the WE normalized the FBGL back to normal and had an even greater hypoglycemic effect than quercetin. The WE was found to be rich in apigenin *C*-glycosides. One apigenin *C*-glycoside (apigenin-6-*C*-β-fructopyranoside) had been reported to possess anti-diabetic properties [34]. The histology study showed that the WE and quercetin both had a desirable effect on the number of functional Langerhans islets in the pancreas, which could partially explain their hypoglycemic effects. The enteritis seen in the CF-treated group could be due to the high concentration of the fraction administered to the mice. The enteritis in this group was associated with a noticeable weight loss that could ultimately explain why the mice in this group did not survive the whole 20 days of the experiment. The WE showed a very promising hypoglycemic effect that justifies the use of this plant as a natural hypoglycemic by the traditional healers in the Middle East.

Plants from the genus *Achillea* are used in the Middle East region not only for their medical benefits, but are also casually consumed as a hot beverage. Because the anti-phlogistic potential of *A. wilhelmsii*, especially the lipophilic fractions, was proven in this work, it is possible to say that casual consumption of this plant as a part of the diet could attenuate the inflammatory state, such as that induced by *Helicobacter pylori* or ethanol-induced gastric ulcers [9,35], and improve the outcome of many inflammation-related disorders. The EAF had the most potent effect among the tested samples. It reduced the TNF-α secretion, attenuated the level of MMP-2 and MMP-9 and was the only fraction to inhibit the digestion of the pro-MMP-2 proenzyme to a mature, biologically-active form.

Persistent high serum levels of cholesterol are a cause of cardiovascular diseases and possible death and contribute to the formation of atherosclerotic plaques in arteries throughout the body. Atherosclerosis is one of the leading causes of deaths out of all = non-communicable diseases, such as cancer, hypertension and diabetes [15]. The atherosclerotic process, together with the development of metabolic syndrome, affects a large number of the adult population worldwide. Pravastatin is a representative of the statin class of drugs that in their active hydrolysed form are specific inhibitors of HMGR. Both 1β,10β-epoxydesacetoxymatricarin (CP1) and leucodin (CP2) showed an inhibition activity comparable to that of pravastatin.

## 4. Materials and Methods

### 4.1. Plant Material

*A. wilhelmsii* air-dried aerial parts were obtained from Syria. The plant was collected from an area close to the Anti-Lebanon Mountains (July 2010). The plant was identified and authenticated by Necmi Aksoy, Department of Forest Botany, Faculty of Forestry, Duzce University, Turkey. The voucher specimen (DUOF No: 1602) was deposited at the herbarium of The Faculty of Forestry (DUOF), Department of Forest Botany, Faculty of Forestry, Duzce University, Turkey. The identification of this plant was confirmed by Jiří Danihelka, Department of Botany and Zoology, Faculty of Science, Masaryk University, Czech Republic.

### 4.2. Extraction

#### 4.2.1. Preparation of *A. wilhelmsii* Crude Extracts

Crushed plant material (500 g) was defatted with n-hexane, dried and then extracted three times with distilled water. The filtrate was combined, freeze-dried and refrigerated (WE). The yield of this was 51.84 g. The plant material was dried again and extracted with a hydro-alcoholic mixture (75% ethanol in water) three times. The filtrate was combined, concentrated under reduced pressure, freeze-dried and refrigerated. The yield of this was 24.33 g.

#### 4.2.2. Fractionation by Solvent-Solvent Extraction

Half of the water extract (WE) was utilized for immiscible liquid-liquid extraction using ethyl acetate. The ethyl acetate fraction (EAF) was collected and dried to give a yield of 2.22 g. Half of the hydro-alcoholic extract (HAE) was also separated into two parts by immiscible liquid-liquid extraction using chloroform. The chloroform fraction (CF) was collected and dried to give a yield of 8.49 g.

### 4.3. Phytochemical Analyses

#### 4.3.1. HPLC-DAD-MS Analysis

The analysis of *A. wilhelmsii* extracts and fractions was performed by reversed-phase LC equipped with DAD and negative ion ESI with MS/MS. LC was performed with an Agilent (Santa Clara, CA, USA) 1100 Series LC system. The data were processed using Agilent Rev.B.04.01 (481) ChemStation. MS detection was performed using an Agilent 1100 LC-MSD Trap system VL series. The gas flow rate of N_2_ was 10 L/min; the capillary voltage was 3.5 kV; the nebulization was pressure 80 psi; and the gas temperature was 300 °C. Spectra were recorded in negative ion mode between *m/z* 150 and 1500. The data were processed using Agilent LC/MSD Trap Software 5.3. A SUPELCOSIL ABZ+PLUS, (3 µm, 15 cm × 4.6 mm) column, thermostated at 40 °C, was used. The solvents were (A) 0.2% formic acid in water and (B) MeCN. The elution gradient was 10% to 100% B in A over 36 min using a flow rate of 1 mL/min. The absorbance was recorded at 215, 230, 254, 280 and 350 nm.

#### 4.3.2. Determination of Phenolic Compounds Content

The total content of polyphenols was determined by a modified Folin-Ciocalteu colorimetric method [25]. A sample at a concentration of 5 mg/mL was dissolved either in water or DMSO depending on its solubility. The results were expressed as gallic acid equivalent (GAE) per gram of dry extract weight. All measurements were done in triplicate.

#### 4.3.3. Determination of Flavone and Flavonol Content

Flavone and flavonol contents were analyzed using a colorimetric method. The method was described in detail in the literature [25]. A sample at a concentration of 5 mg/mL was dissolved either in water or DMSO depending on its solubility. The results were expressed as quercetin equivalent (QE) per gram of dry extract weight. All measurements were done in triplicate.

### 4.4. The Hypoglycemic Activity

#### 4.4.1. Animals

Adult male mice CD1 (standard laboratory mouse) weighing 20 to 25 g purchased from Masaryk University (Brno, Czech Republic, 30048/2007-10001, number: CZ-62760157) were used. The mice were housed in an air-conditioned animal laboratory under standard conditions; that is, a temperature of 22 °C, a relative humidity of 50% and a 12-h light/dark cycle. The animals were allowed to acclimatize for 5 days and were fed with a pellet diet and tap water *ad libitum*.

#### 4.4.2. Induction of Experimental Diabetes

Alloxan monohydrate (Sigma-Aldrich, Munich, Germany) was freshly dissolved in sterile normal saline and injected intravenously (120 mg/kg body weight) in the tail vein of the test subjects. Two days after the administration of alloxan, fasting blood glucose levels (FBGL) were measured using One Touch Ultra Easy glucometer (Johnson & Johnson, division LifeScan, New Brunswick, NJ, USA), and diabetic mice with levels higher than 20 mmol/L were selected and distributed into 7 groups randomly (6 mice in each group). All aspects of animal care complied with the ethical guidelines and technical requirements and were proven to be consistent with the Animal Scientific Procedures Act 86/609/EC. The state of health of all animals was regularly examined several times a day during both the period of the animal’s acclimation and the whole course of the experiment, by the working team whose members are holders of the Certificate on Professional Competence issued by the Central Commission for the Animal Protection pursuant to § 17 of the Act on Protection of Animals against Cruelty (No. 246/1992 Collection) of the Czech National Council.

#### 4.4.3. Experimental Design

A total of 42 mice (6 mice in each group) were used. The plant extracts and fractions were prepared by dissolving the samples in normal saline prior to use. Kolliphor EL (Sigma-Aldrich) was used to solubilize the HAE, EAF and CF in percentages of 3%, 2% and 5%, respectively. The quercetin suspension was prepared by suspending quercetin in saline using 3% Kolliphor EL and used as a standard for comparison. These prepared solutions were administered by gavage (i.g.) to the groups for 20 consecutive days according to the following:

Group 1: Healthy mice;
Group 2: Diabetic control;
Group 3: Diabetic mice + 200 mg/kg WE;
Group 4: Diabetic mice + 200 mg/kg EAF;
Group 5: Diabetic mice + 200 mg/kg HAE;
Group 6: Diabetic mice + 200 mg/kg CF;
Group 7: Diabetic mice + 50 mg/kg quercetin suspension.

#### 4.4.4. Histology Study

The mice were sacrificed by cervical dislocation. Immediately after, samples of the pancreas, liver, kidney and gut were collected and fixed in 10% buffered formaldehyde (PH 7.2–7.4). Samples were further dehydrated by an ascending ethanol line (30%, 50%, 70%, 80%, 95%, 100%), lightened by xylene and fixed in paraffin. Paraffin blocks were cut using a microtome (Leica SM 2000, Prague, Czech Republic) into sections of 3.5 µm and stained by hematoxylin-eosine pigment.

### 4.5. The Effect upon Inflammatory Mediators

#### 4.5.1. Materials

The RPMI 1640 medium and the penicillin-streptomycin mixture were purchased from Lonza (Brussel, Belgium). Fetal bovine serum, phorbol myristate acetate, prednisone and the LPS obtained from *Escherichia coli* 0111:B4 were purchased from Sigma-Aldrich. Instant ELISA Kits (eBioscience, Vienna, Austria) were used to evaluate the production of TNF-α.

#### 4.5.2. Cell Maintenance and Macrophage Preparation

The human monocytic leukemia cell line THP-1 was obtained from the European Collection of Cell Cultures (ECACC, Salisbury, UK). The cells were cultivated at 37 °C in an RPMI 1640 medium supplemented with 2 mM l-glutamine, 10% fetal bovine serum, 100 U/mL of penicillin and 100 µg/mL of streptomycin in a humidified atmosphere containing 5% CO_2_. Monocytes’ differentiation to macrophages was induced by phorbol myristate acetate [36].

#### 4.5.3. Drug Treatment, Induction of Inflammation and Evaluation of TNF-α Secretion

Differentiated macrophages were pretreated for 1 hour with *A. wilhelmsii* extracts and fractions dissolved in either water or DMSO to obtain a final concentration of 25 μg/mL (this concentration lacked the cytotoxic effect). For comparison, 1 μM prednisone dissolved in DMSO was used as a standard. Vehicle-treated cells contained a vehicle (DMSO) only, and control cells were without the lipopolysaccharide treatment. The concentration of DMSO was 0.1% in each well. The inflammatory response was triggered by adding the lipopolysaccharide dissolved in water (1 µg/mL) to the drug-pretreated macrophages, and the cells were incubated another 24 h. After this time period, the medium was collected, and the concentration of TNF-α was measured. The lowest detection limit was 7.8 pg/mL of TNF-α.

#### 4.5.4. Zymography

Conditioned media obtained by the same way as for TNF-α evaluation were used for measurement of matrix-metalloproteinases (MMP) activity by zymography [37]. Briefly, 20 μL of collected medium were loaded onto polyacrylamide gel impregnated with 0.1% gelatin. After electrophoresis, the SDS from the gels was washed out by 2.5% (*v*/*v*) Triton X100, and the gels were incubated for 30 min at room temperature (23 °C) and overnight (16 to 20 h) at 37 °C in the developing buffer (50 mM Tris pH 8.8, 5 mM CaCl_2_, 3 mM NaN_3_ and 0.5% Triton ×100). Gels were stained by Coomassie blue. The intensity of digested regions was calculated by AlphaEasy FC 4.0.0 software (Alpha Innotech, San Leandro, CA, USA) for densitometric analysis.

### 4.6. Statistical Analysis

Statistical analysis was performed by one-way analysis of variance using GraphPad Prism (Version 5, GraphPad, La Jolla, CA, USA). The significant differences were assessed with Tukey’s honestly significant difference test (*p* < 0.05). For the effect upon inflammatory mediators assay, statistical significance was determined at levels of *p* < 0.05, *p* < 0.01 and *p* < 0.001.

### 4.7. The Anti-Hypercholesterolemic Activity

#### 4.7.1. *In Vitro* HMGR Inhibition Assay

The commercially available HMGR assay kit from Sigma-Aldrich, Catalog Number CS1090, was used to evaluate the HMGR inhibition according to the manufacturer’s instructions. The concentration of the purified human enzyme stock solution (Sigma) was 0.50 to 0.70 mg protein/mL. Reference statin drug pravastatin (from Sigma) was used as the positive control. To characterize HMGR inhibition under defined assay conditions, reactions containing 4 µL of NADPH and 12 µL of HMG-CoA substrate in a final volume of 0.2 mL of 100 mM potassium phosphate buffer, pH 7.4, were initiated (Time 0) by the addition of 2 µL of the catalytic domain of human recombinant HMGA incubated in BioTek Synrgy HT (Winooski, VT, USA) at 37 °C in the presence or absence (control) aliquots of the tested samples dissolved in water or DMSO. The blank experiment did not contain HMG-CoA reductase nor any of the studied substances. The rates of NADPH consumed were monitored every 20 s for up to 10 min by scanning spectrophotometrically.

A 2.5-mg/mL concentration of each extract or fraction was used for the measurement. Two microliters were the amount of the sample used in the well. All measurements were done in triplicate. The percentage of inhibition was calculated as follows:

$$\%\text{ Inhibition}=\frac{\Delta A340\text{control }–\;\Delta A340\text{sample}}{\Delta A340\text{control}}\times 100\%$$

#### 4.7.2. The Isolation, Identification and HMGR Inhibition Activity Testing of the Main Compounds in the CF

The main 5 compounds (Peaks 3, 4, 5, 6 and 7) in the CF were separated using reversed-phase preparative HPLC on an YL 9100 HPLC System (Young Lin, Korea) with a Foxy R2 Fraction Collector (Teledyne Isco, Lincoln, NE, USA). The column was SUPELCOSIL RP-amide, 250 cm × 10 mm, particle size 5 μm. Gradient elution employed 0.2% HCOOH and MeCN, in the gradient: initial composition 30% MeCN, final composition 100% MeCN in the 30th min; flow rate 5 mL/min. Fractions were acquired according to the detector response at λ = 254 nm. The purity of all compounds exceeded 95%, as checked via analytical HPLC.

The identity of the compounds was validated using nuclear magnetic resonance NMR (^1^H-NMR) (Billerica, MA, USA). The carbon chemical shifts were obtained by NMR (HSQC and HMBC) spectra that were obtained on a Bruker Avance 400 spectrometer (Billerica, MA, USA) with TMS as an internal standard. HRMS were measured using an ESI-TOF spectrometer (Mariner Biosystems, Applied Biosystems, Waltham, MA, USA) using ESI in the positive mode of ionization.

The 5 isolated compounds (1β,10β-epoxydesacetoxymatricarin (CP1), leucodin (CP2), 5-demethylsinensetin (CP3), 2-(3,4-dimethoxy-phenyl)-3-hydroxy-5,6,7-trimethoxy-chromen-4-one (CP4) and salvigenin (CP5)) were evaluated for their HMGR inhibition activity using the same protocol. The compounds were tested at 0, 2.5, 5, 10, 20, 40 and 80 µM. All measurements were done in triplicate. All results were expressed as the mean ± SD of the three repetitions, and IC_50_ values, *i.e.*, the half maximal inhibition concentration, were calculated using GraphPad Prism software.

#### 4.7.3. Computational Docking

##### Ligand Preparation

All ligands were modelled using Marvin 15.4.20.0, 2015, ChemAxon, and the conformer with the lowest potential energy was used as an input to PyRX 0.8. Ligands were then prepared using PyRX with default settings.

##### Protein Preparation

PyRX was also used for the protein preparation with default settings. The crystal structure of human HMG-CoA reductase inhibited by atorvastatin (PDB ID: 1HWK) was downloaded from the Research Collaboratory for Structural Bioinformatics Protein Data Bank (http://www.rcsb.org/pdb/home/home.do) [38]. Extraneous atoms, alternate amino acid residue conformations, ligands, ions and solvent molecules were removed. The protein was then prepared using the PyRX program with default settings.

##### Molecular Docking

PyRX was used in conjunction with AutoDock Vina [39]. As a binding site, we used the position of atorvastatin determined by the crystallographic experiment. Search exhaustiveness for AutoDock Vina was set to 30, and the edge of the cube defining the searching space was set to 30 Angstroms. The first 10 best solutions according to the binding affinity were stored. For the graphical evaluation of the results, PyMOL was used, and the best solution, ranked by binding affinity, was chosen.

The compounds screened were in accordance with Lipinski‘s rule of five, a compound having not more than 5 hydrogen bond donors (OH and NH groups), not more than 10 hydrogen bond acceptors (notably N and O), molecular weight under 500 g/mol, partition coefficient log P of less than 5 and rotatable bonds of less than 10.

## Figures and Tables

**Table 1:** Total content of polyphenols, flavones and flavonols in the studied extracts and fractions. Each value represents the mean ± SD of three independent measurements.

Sample	Total Content of Phenolic Compounds (GAE mg/g)	Total Content of Flavones and Flavonols (QE mg/g)
WE	33.11 ± 1.12	3.64 ± 0.21
EAF	104.75 ± 6.12	11.51 ± 0.85
HAE	55.77 ± 2.94	11.28 ± 1.05
CF	46.40 ± 2.22	13.27 ± 0.45

**Table 2:** The *in vivo* hypoglycemic activity assay results for the tested groups. Each value is the mean ± SD for six mice in each group. The empty cells in the table are because the mice in the CF-treated group did not survive past Day 15.

Day	Healthy Group	Diabetic Group	EAF-Treated Group	HAE-Treated Group	CF-Treated Group	WE-Treated Group	Quercetin-Treated Group
	FBGL (mmol/L)	FBGL (mmol/L)	FBGL (mmol/L)	FBGL (mmol/L)	FBGL (mmol/L)	FBGL (mmol/L)	FBGL (mmol/L)
0	6.00 ± 0.84 ^a^	30.80 ± 2.65 ^b^	30.11 ± 2.84	30.27 ± 1.88	31.23 ± 1.19	30.71 ± 2.83	29.63 ± 2.67
4	5.67 ± 1.11 ^a^	30.69 ± 1.97 ^b^	24.71 ± 2.99 ^a^	29.58 ± 1.94	33.45 ± 1.55	28.13 ± 2.93	27.58 ± 2.09 ^a^
8	5.75 ± 1.05 ^a^	29.46 ± 2.74 ^b^	20.07 ± 1.95 ^a^	26.27 ± 2.17 ^a^	32.00 ± 2.25	15.18 ± 2.89 ^a^	21.02 ± 1.63 ^a^
12	6.35 ± 0.99 ^a^	29.13 ± 1.98 ^b^	18.04 ± 2.79 ^a^	21.22 ± 1.45 ^a^	34.35 ± 1.06	12.73 ± 2.16 ^a^	18.70 ± 1.26 ^a^
16	6.12 ± 0.90 ^a^	30.46 ± 2.41 ^b^	13.19 ±2 .22 ^a^	16.77 ± 1.46 ^a^	-	8.10 ± 2.48 ^a^	17.55 ± 1.61 ^a^
20	5.85 ± 0.97 ^a^	31.09 ± 2.06 ^b^	12.40 ± 2.11 ^a^	14.08 ± 1.34 ^a^	-	4.39 ± 1.54 ^a^	14.02 ± 2.06 ^a^

^a^ The number is statistically significant when compared to the diabetic group (the comparison is between the readings taken on the same day) (*p* < 0.05). ^b^ The number is statistically significant when compared to the healthy group (the comparison is between the readings taken on the same day) (*p* < 0.05).

**Table 3:** HMGR percentage inhibition values for the extracts and fractions of *A. wilhelmsii,* along with the control (pravastatin). Each value represents the mean ± SD of three independent measurements.

Sample	HMGR Inhibition (%)
WE	36.11 ± 4.21
EAF	64.70 ± 2.50
HAE	70.15 ± 3.84
CF	89.21 ± 7.24
Pravastatin	98.02 ± 3.34

**Table 4:** The binding affinity of different ligands (the 5 compounds and 2 selected statins).

Ligand	Binding Affinity (Kcal/mol)
1β,10β-epoxydesacetoxymatricarin (CP1)	−7.6
Leucodin (CP2)	−8.0
5-demethylsinensetin (CP3)	−7.5
2-(3,4-dimethoxy-phenyl)-3-hydroxy-5,6,7-trimethoxy-chromen-4-one (CP4)	−7.4
Salvigenin (CP5)	−7.4
Atorvastatin	−9.6
Pravastatin	−7.0

## Supplementary Materials

Supplementary materials can be accessed at: http://www.mdpi.com/1420-3049/21/4/404/s1.

## Abbreviations

The following abbreviations are used in this manuscript:

*A. wilhelmsii*	*Achillea wilhelmsii*
CF	chloroform fraction
CP1	1β,10β-epoxydesacetoxymatricarin
CP2	Leucodin
CP3	5-demethylsinensetin
CP4	2-(3,4-dimethoxy-phenyl)-3-hydroxy-5,6,7-trimethoxy-chromen-4-one
CP5	Salvigenin
DUOF	Herbarium of the Faculty of Forestry
EAF	ethyl acetate fraction
FBGL	fasting blood glucose levels
GAE	gallic acid equivalent
HAE	hydro-alcoholic extract
HMGR	3-hydroxy-3-methylglutaryl-CoA reductase
MMP	matrix-metalloproteinases
QE	quercetin equivalent
THP-1	human monocytic cell line derived from an acute monocytic leukemia patient
TNF-α	tumor necrosis factor α
WE	water extract
