# Supplementary material for: The Chemical Composition of Achillea wilhelmsii C. Koch and Its Desirable Effects on Hyperglycemia, Inflammatory Mediators and Hypercholesterolemia as Risk Factors for Cardiometabolic Disease
Source: Molecules. 2016 Mar 25;21(4):404. doi: 10.3390/molecules21040404 (PMC6273470; doi:10.3390/molecules21040404)
Supplement: Supplementary file 1 [file molecules-21-00404-s001.pdf]

Supplementary Materials: The Chemical Composition of *Achillea wilhelmsii* C. Koch and Its Desirable Effects on Hyperglycemia, Inflammatory Mediators and Hypercholesterolemia As Risk Factors for Cardiometaboic Disease

Elian Khazneh, Petra Hřibová, Jan Hošek, Pavel Suchý, Peter Kollár, Gabriela Pražanová, Jan Muselík, Zuzana Hanaková, Jiří Václavík, Michał Miłek, Jaroslav Legáth and Karel Šmejkal

1. Structure and spectral data of 1β,10β-epoxydesacetoxymatricarin (CP1)

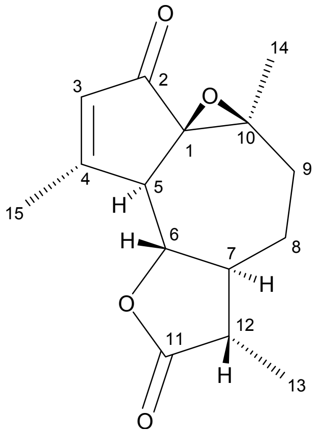

1β,10β-epoxydesacetoxymatricarin (CP1)

$M_W = 262$

$[\alpha]_D^{25} = -9.4$ (c = 0.2 g/100 mL, MeOH)

UV (MeOH) λ_{max} (log ε): 234 (4.03) nm

HRMS *m*/*z* 263.1288 (calcd for $C_{15}H_{18}O_4^+$, 263.1283)

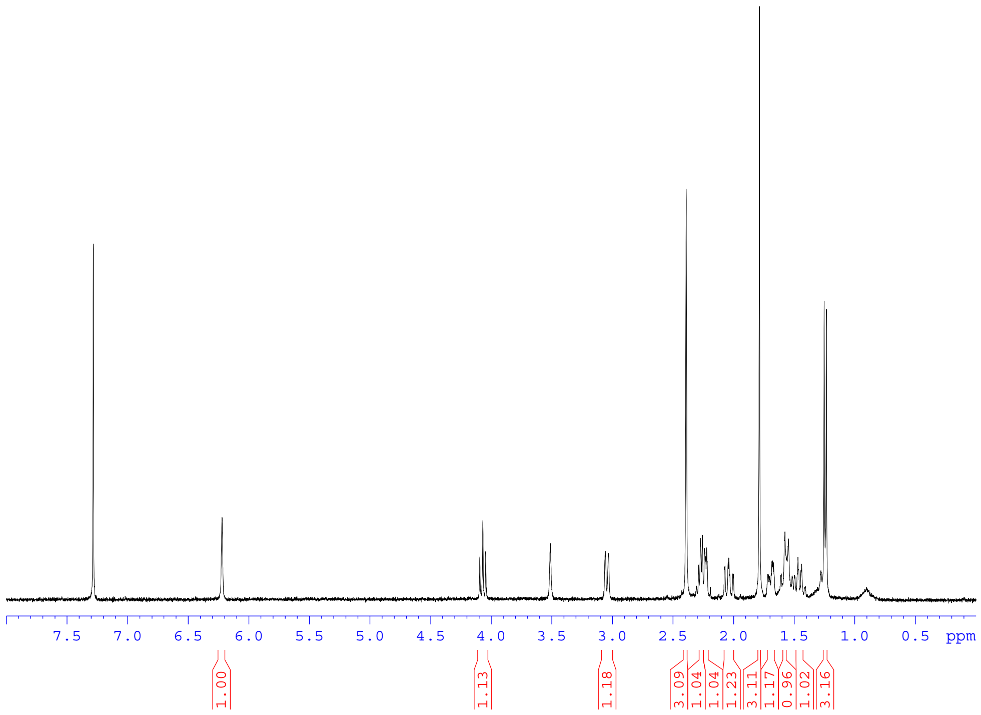

Figure S1. ^{1}H-NMR spectrum for CP1.

Table S1. Chemical shifts (δ in ppm) for CP1; Solvent: $CDCl_3$.

Position	δ_C	δ_H (*J* in Hz)	HMBC
1	66.7, C		
2	200.8, C=O		
3	133.2, CH	6.22, s	1, 2, 4, 5, 15
4	176.5, C		
5	49.2, CH	3.05, d (10.0)	1, 3, 4, 6, 7
6	80.3, CH	4.08, t (10.0)	1
7	56.1, CH	1.47, m (2.5, 10.0)	-
8	21.7, CH_2	1.57, brd (11.7)	7, 9, 10
		1.70, m (2.5)	7, 9, 10
9	34.6, CH_2	2.06, dd (11.7, 16.5)	7, 8, 10, 14
		2.25, brd	7, 8, 10, 14
10	65.2, C		
11	41.3, CH	2.27, dq (2.5, 7.0)	7, 12, 13
12	178.8, C=O		
13	12.2, CH_3	1.25, d (7.0)	7, 11, 12
14	18.8, CH_3	1.78, s	1, 9, 10
15	20.6, CH_3	2.39, s	3, 4, 5

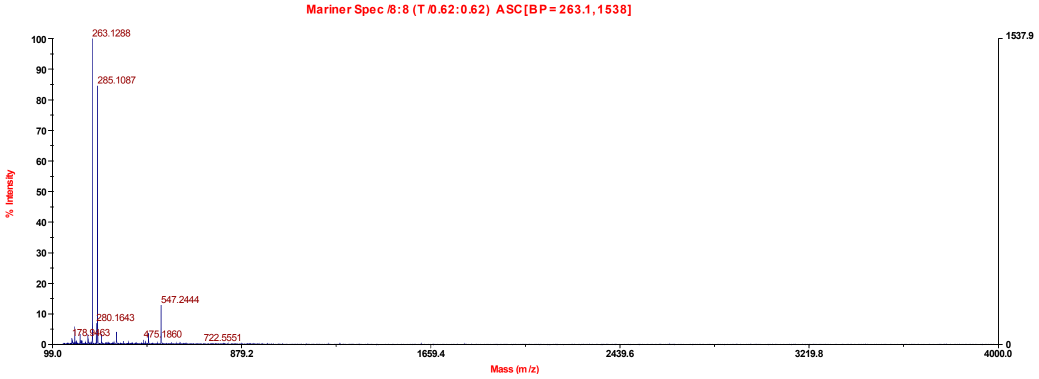

Figure S2. HDMS spectrum for CP1.

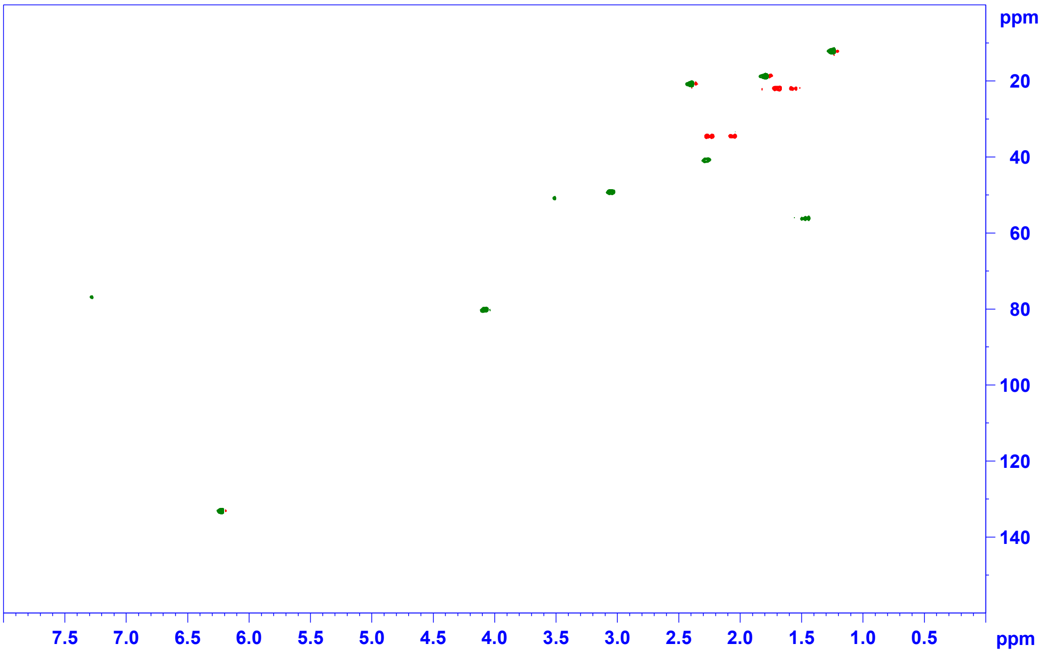

Figure S3. HSQC spectrum for CP1.

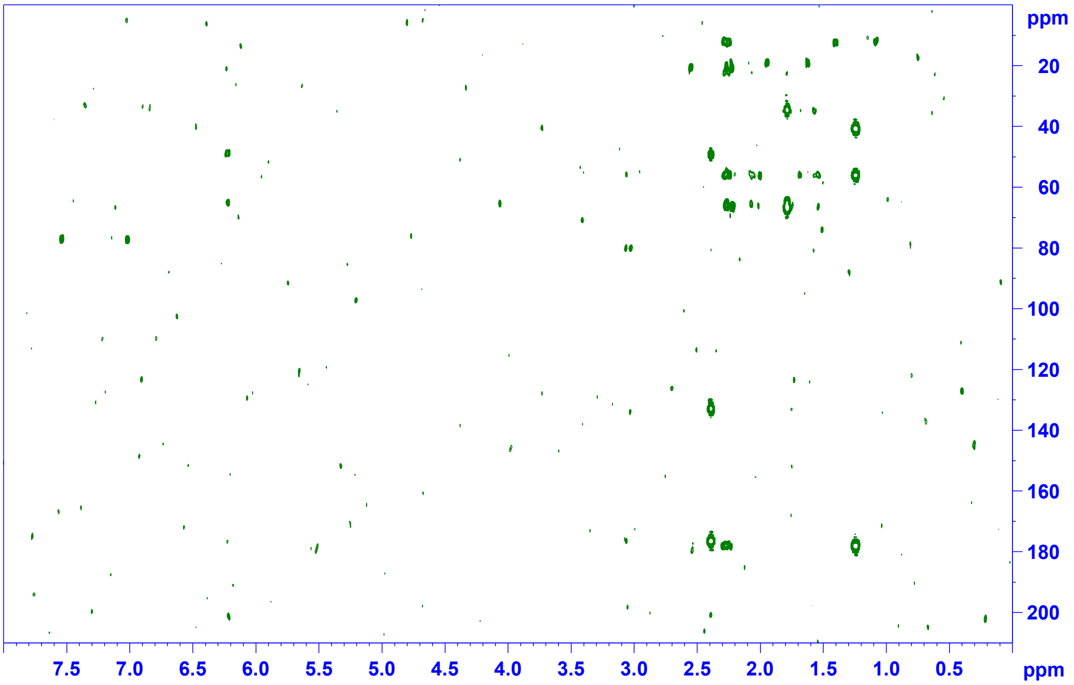

Figure S4. HMBC spectrum for CP1.

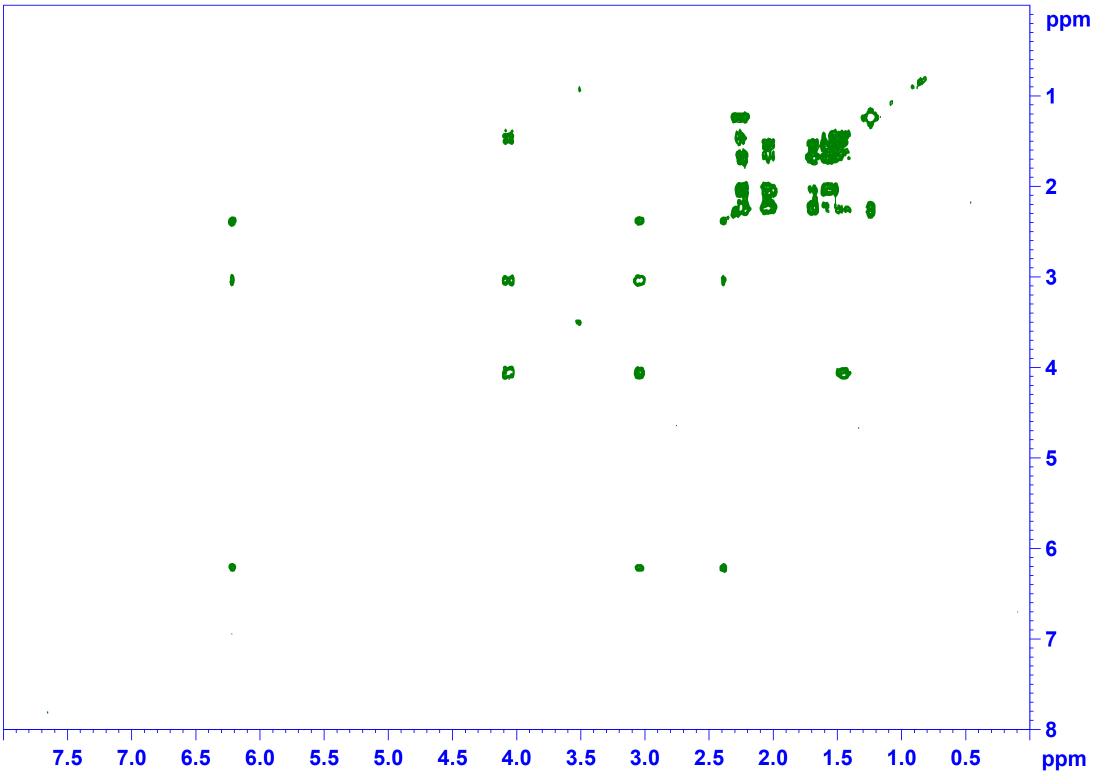

Figure S5. COSY spectrum for CP1.

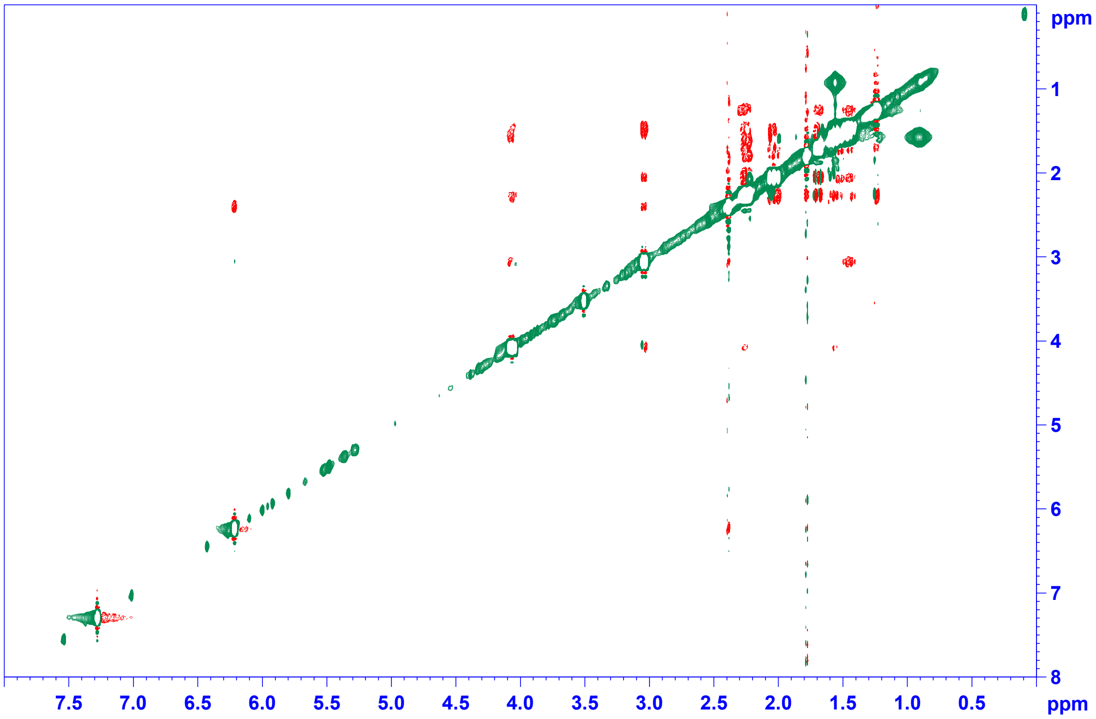

Figure S6. NOESY spectrum for CP1.

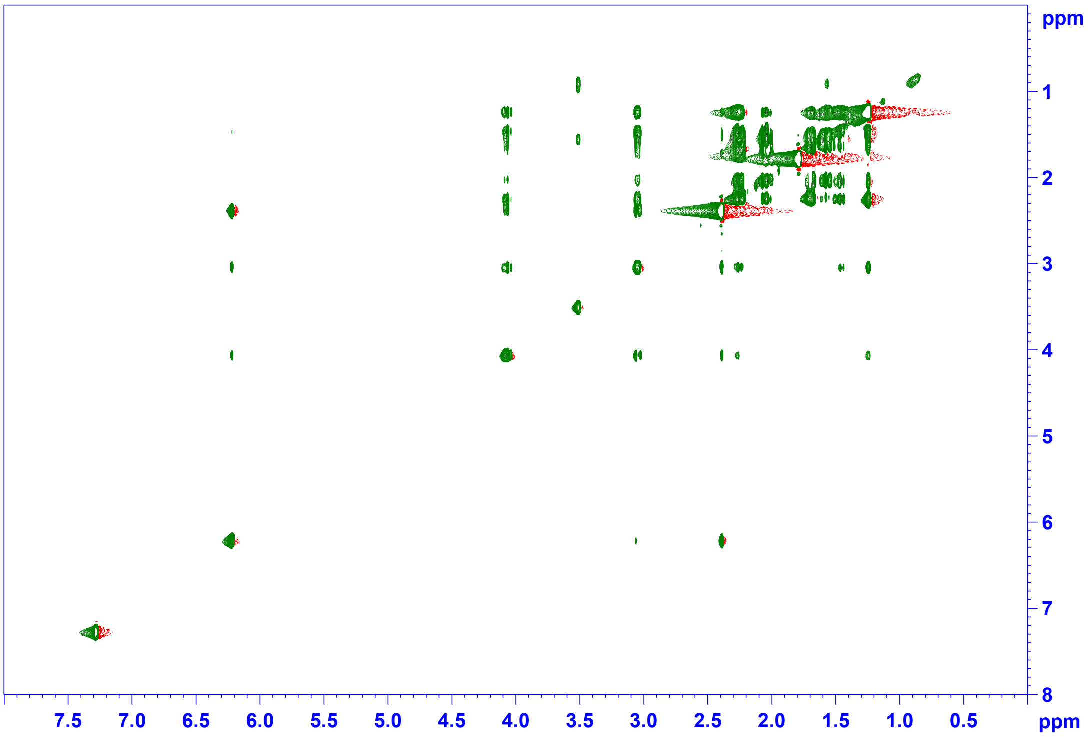

Figure S7. TOCSY spectrum for CP1.

2. Structure and spectral data of Leucodin (CP2)

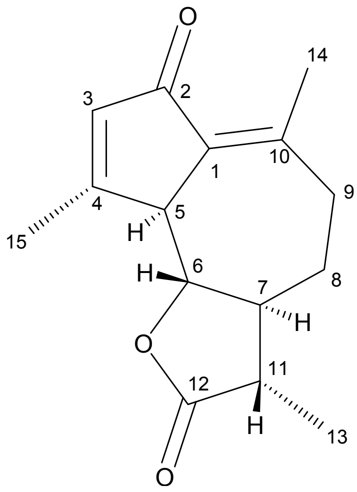

Desacetoxymatricarin (Leucodin) (CP2)

M_W = 246

$[\alpha]_D^{25}$ = + 40.2 (c = 0.2 g/100 mL, MeOH)

UV (MeOH) λ_{max} (log ε): 255 (4.15) nm

MS *m*/*z* (positive mode, APCI)

HRMS *m*/*z* 247.1333 (calcd for $C_{15}H_{18}O_3^+$, 247.1334)

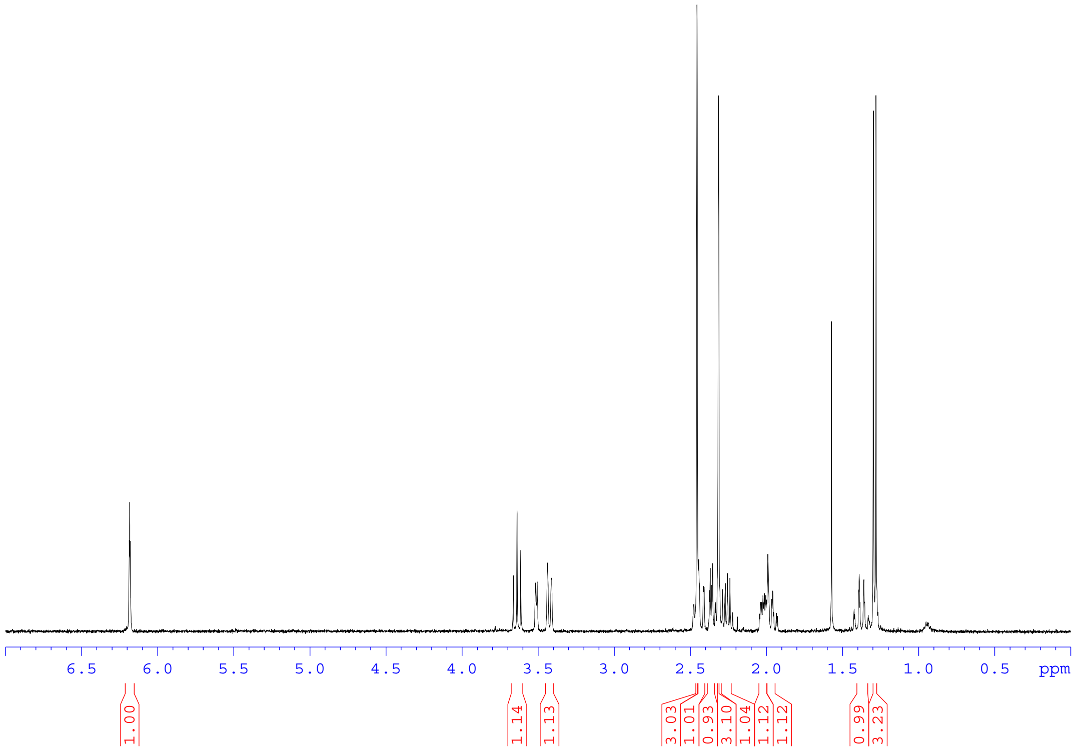

Figure S8. ^{1}H-NMR spectrum for CP2.

Table S2. Chemical shifts (δ in ppm) for CP2; Solvent: $CDCl_3$.

Position	δ_C	δ_H (*J* in Hz)	HMBC
1	131.6, C		
2	196.2, C=O		
3	135.6, CH	6.18, s	1, 2, 4, 5, 15
4	169.0, C		
5	52.4, CH	3.43, d (10.0)	1, 3, 4, 6, 7
6	84.4, CH	3.64, t (10.0)	1, 8, 11
7	56.2, CH	1.97, m (~13.0)	5, 6, 9, 11
8	26.0, CH_2	1.37, m (~13.0)	6, 7, 9, 10
		2.02, m (~13.0)	6, 7, 9, 10
9	37.1, CH_2	2.36, dd (2.0, 6.0)	1, 7, 8, 10, 14
		2.44, dd (2.0, 13.0)	1, 7, 8, 10, 14
10	151.6, C		
11	41.2, CH	2.26, m (7.0)	7, 8, 12, 13
12	177.9, C=O		
13	12.4, CH_3	1.29, d (7.0)	7, 11, 12
14	21.5, CH_3	2.46, s	1, 9, 10
15	19.6, CH_3	2.31, s	3, 4, 5

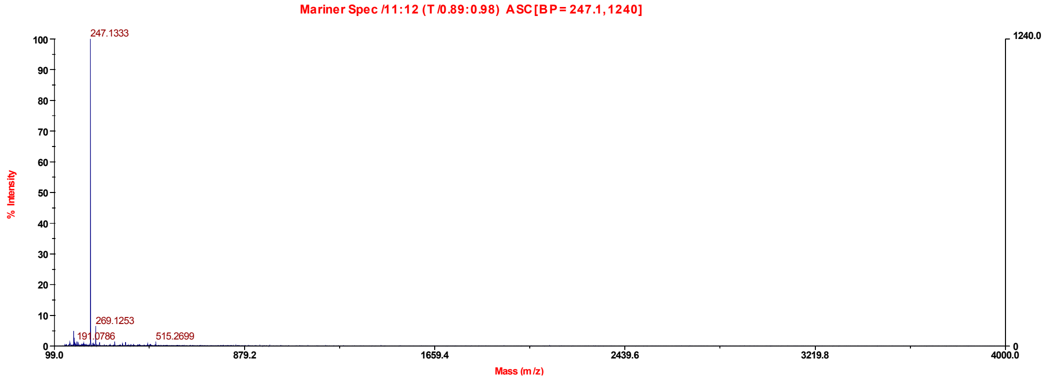

Figure S9. HDMS spectrum for CP2.

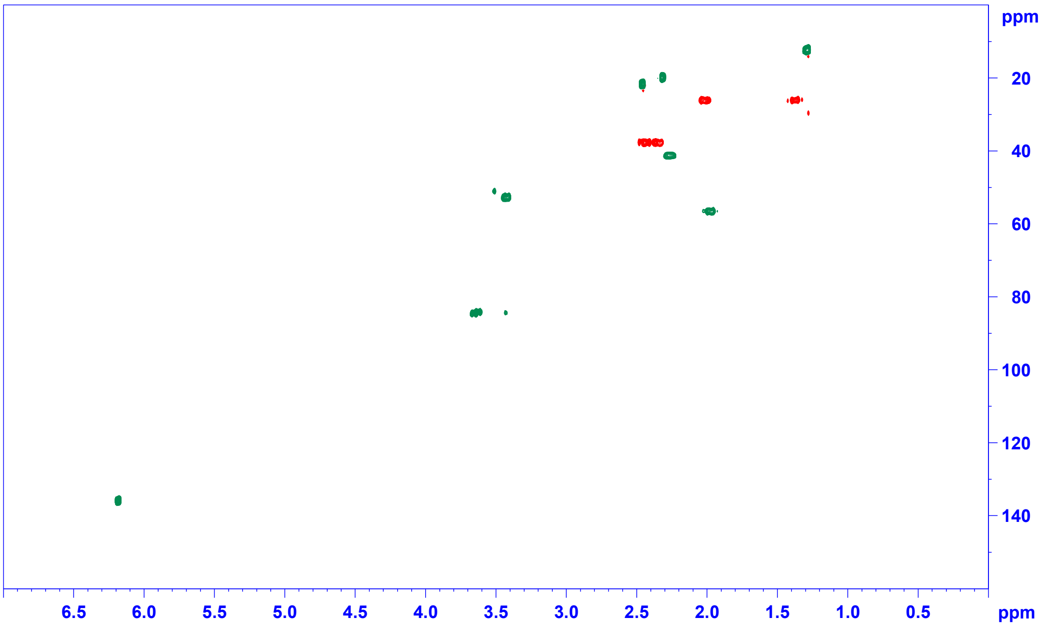

Figure S10. HSQC spectrum for CP2.

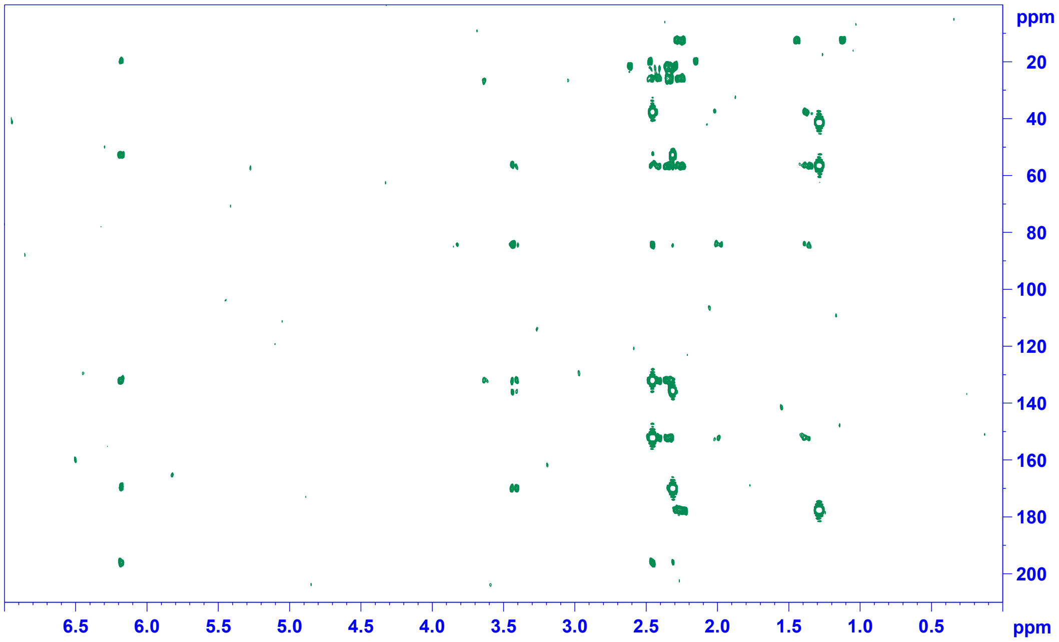

Figure S11. HMBC spectrum for CP2.

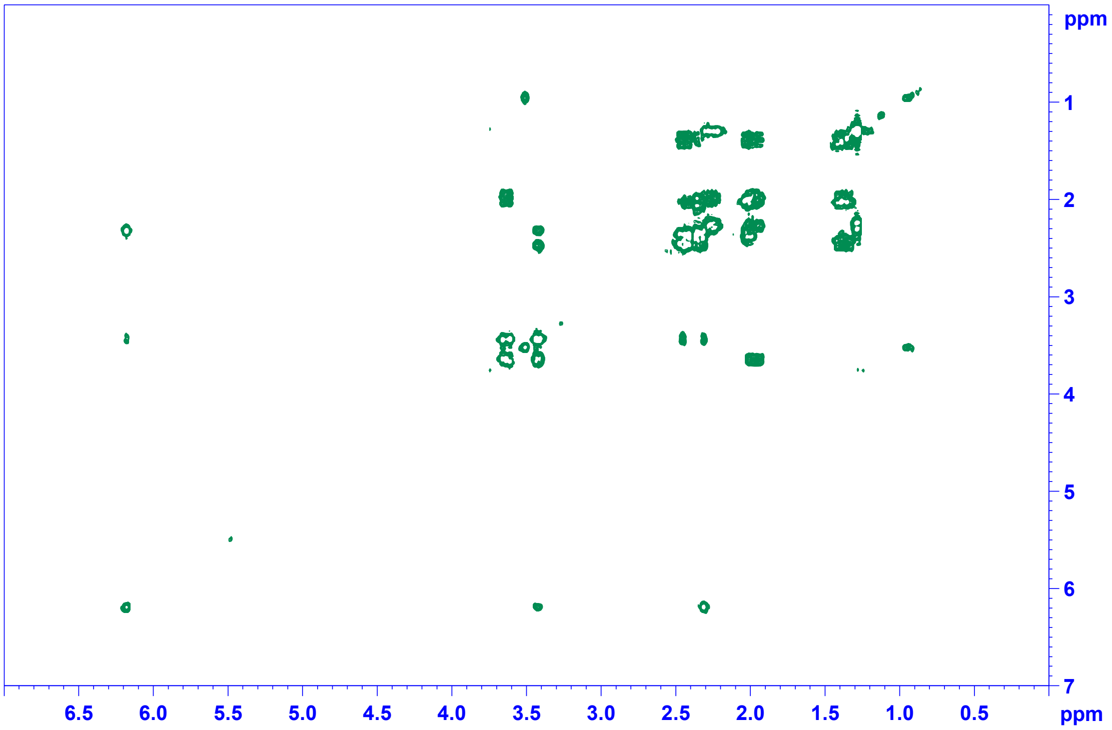

Figure S12. COSY spectrum for CP2.

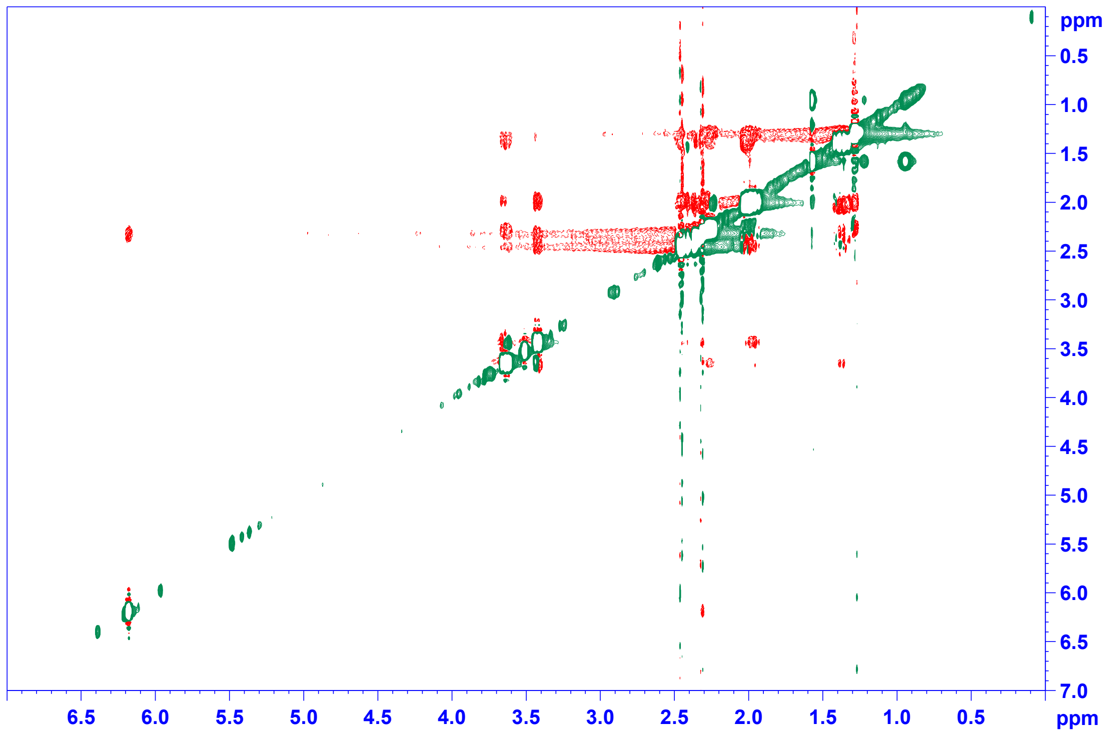

Figure S13. NOESY spectrum for CP2.

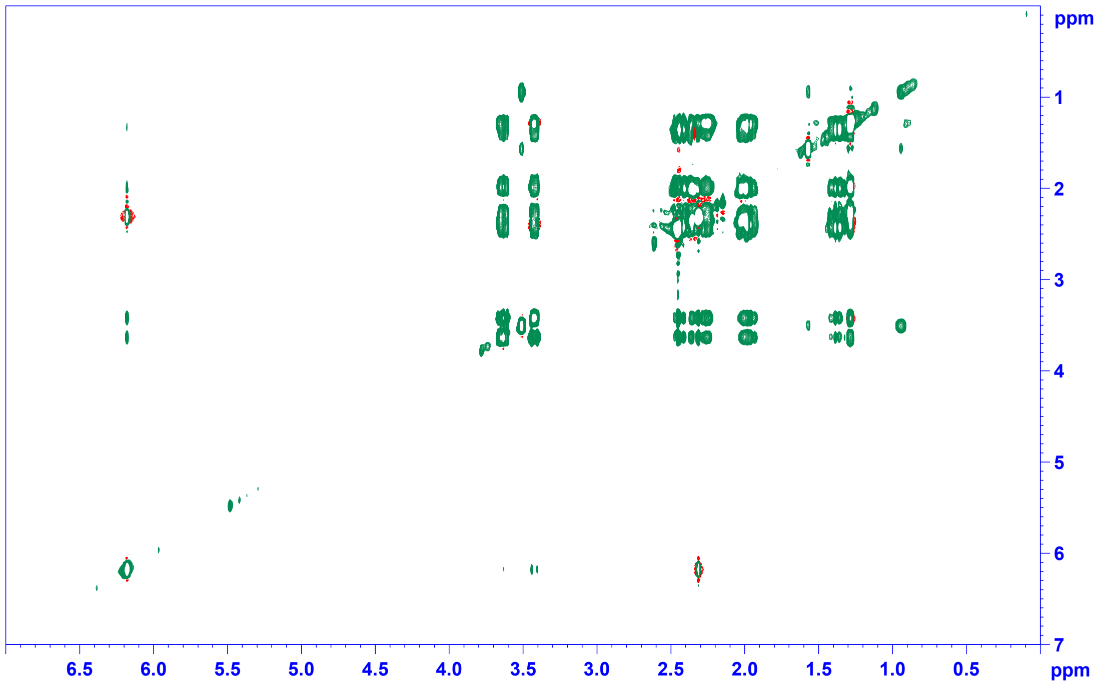

Figure S14. TOCSY spectrum for CP2.

3. Structure and spectral data of 5-demethylsinensetin (CP3)

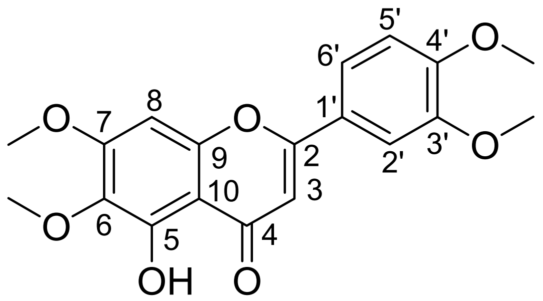

2-(3,4-dimethoxyphenyl)-5-hydroxy-6,7-dimethoxychromen-4-one (5-demethylsinensetin)

M_W = 358

MS *m/z* (positive mode, APCI)

HRMS *m/z* 359.1119 (calcd for $C_{19}H_{18}O_7^+$, 359.1131)

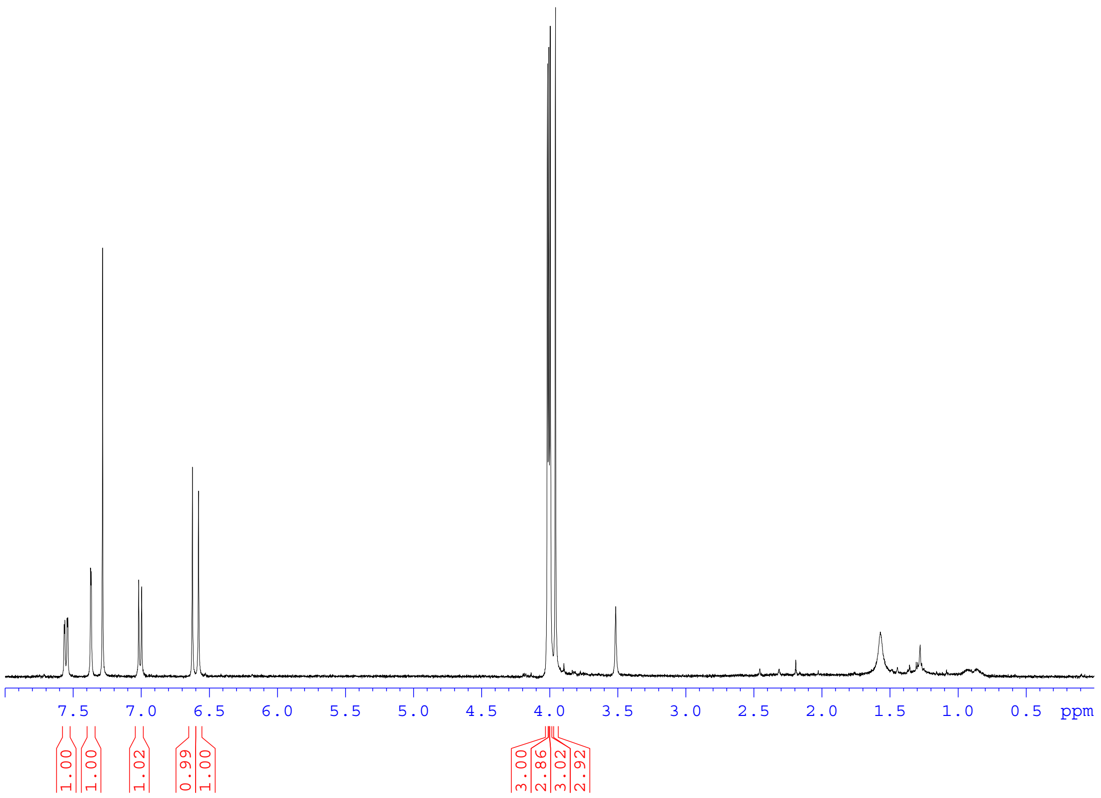

Figure S15. ^{1}H-NMR spectrum for CP3.

Table S3. Chemical shifts (δ in ppm) for CP3; Solvent: $CDCl_3$.

Position	δ_C	δ_H (*J* in Hz)	HMBC
2	163.9, C		
3	104.3, CH	6.63, s	2, 4, 10, 1′
4	182.4, C=O		
5	*not seen*		
6	132.1, C		
7	159.1, C		
8	90.4, CH	6.58, s	4, 6, 7, 9, 10
9	153.7, C		
10	105.7, C		
1′	123.5, C		
2′	108.8, CH	7.37, d (2.0)	2, 3′, 4′, 6′
3′	149.2, C		
4′	152.5, C		
5′	111.2, CH	7.01, d (8.3)	1′, 3′, 4′
6′	120.1, CH	7.55, dd (2.0, 8.3)	2, 2′, 4′
MeO-3′	56.1, CH_3	4.02, s	3′
MeO-4′	56.1, CH_3	3.99, s	4′
MeO-6	60.9, CH_3	3.96, s	6
MeO-7	56.1, CH_3	4.01, s	7

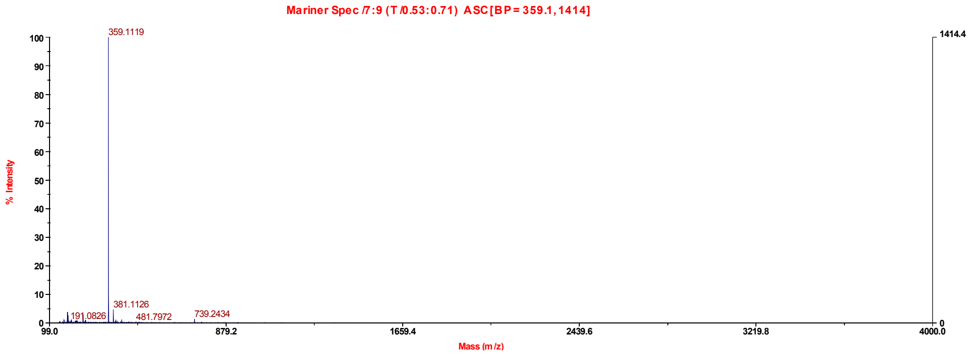

Figure S16. HDMS spectrum for CP3.

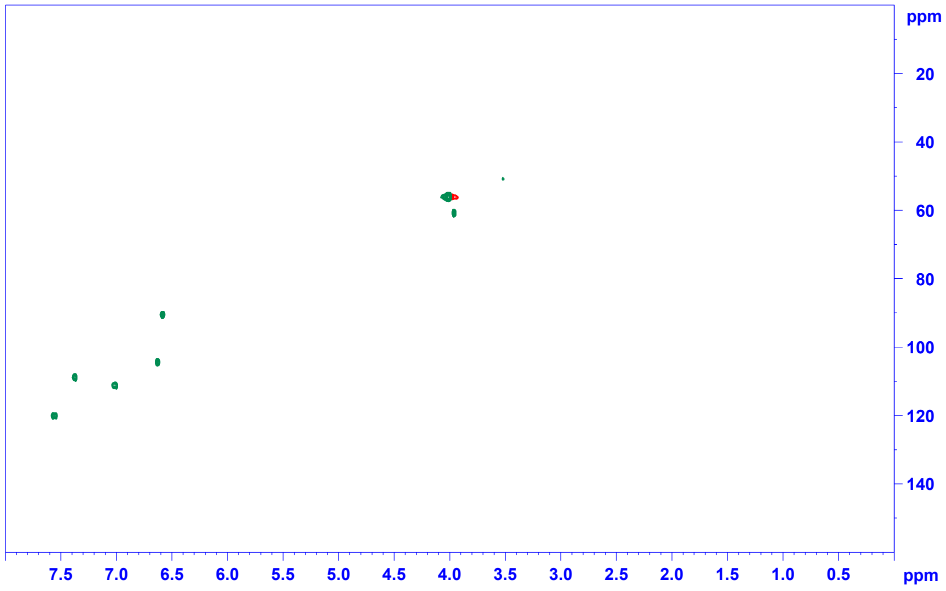

Figure S17. HSQC spectrum for CP3.

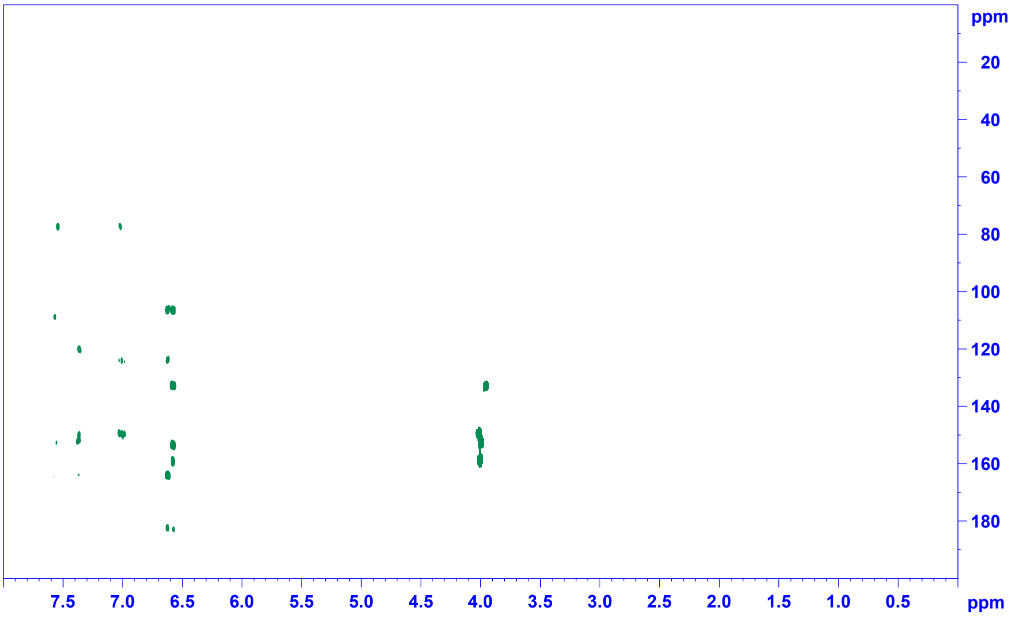

Figure S18. HMBC spectrum for CP3.

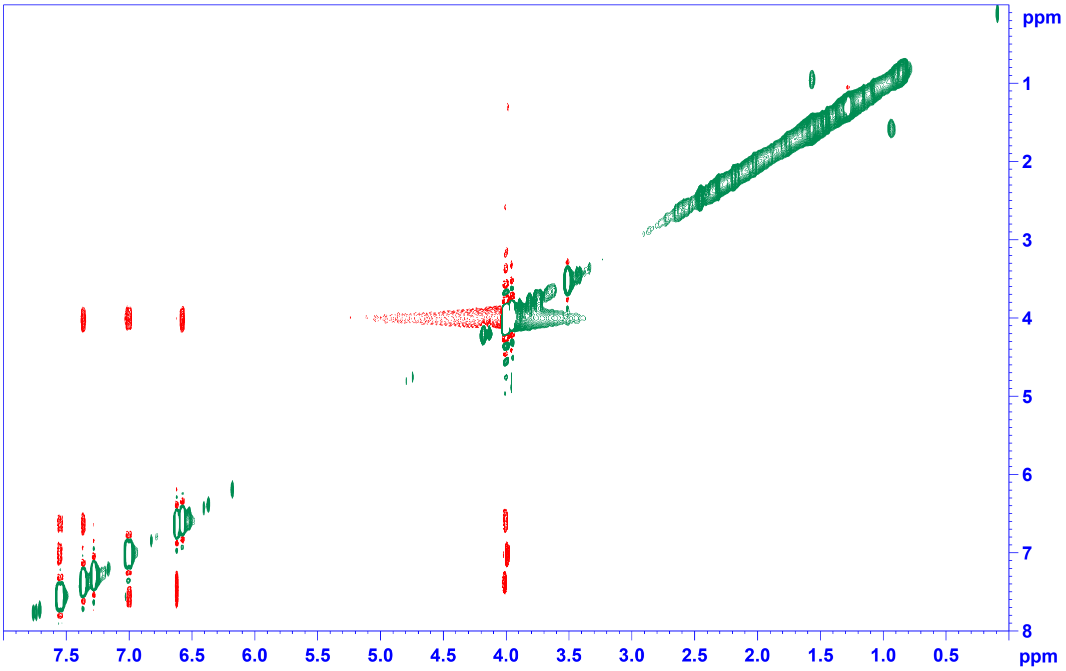

Figure S19. COSY spectrum for CP3.

4. Structure and spectral data of 2-(3,4-dimethoxy-phenyl)-3-hydroxy-5,6,7-trimethoxy-chromen-4-one (CP4)

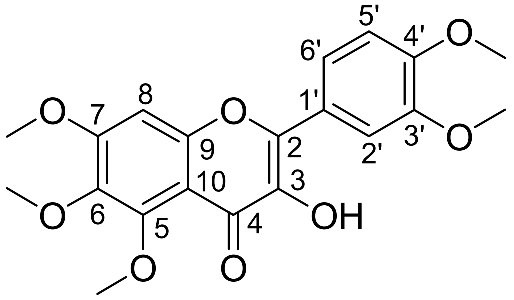

2-(3,4-dimethoxy-phenyl)-3-hydroxy-5,6,7-trimethoxy-chromen-4-one (CP4)

M_W = 388

MS *m*/*z* (positive mode, APCI)

HRMS *m*/*z* 389.1245 (calcd for $C_{20}H_{20}O_8^+$, 389.1236)

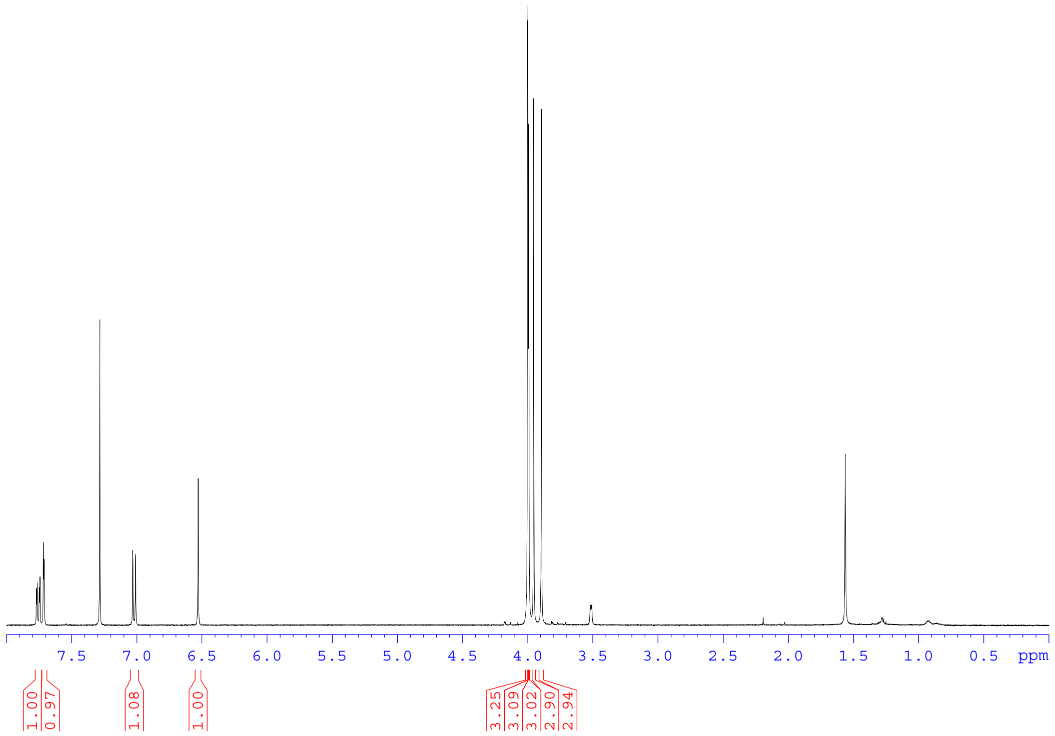

Figure S20. ^{1}H-NMR spectrum for CP4.

Table S4. Chemical shifts (δ in ppm) for CP4; Solvent: $CDCl_3$.

Position	δ_C	δ_H (*J* in Hz)	HMBC
2	155.6, C		
3	*not seen*		
4	179.2, C=O		
5	138.9, C		
6	132.4, C		
7	158.7, C		
8	90.3, CH	6.53, s	4, 6, 7, 9, 10
9	152.1, C		
10	106.4, C		
1′	122.9, C		
2′	111.4, CH	7.71, d (2.0)	2, 4′, 6′
3′	148.7, C		
4′	151.4, C		
5′	110.9, CH	7.02, d (8.5)	1′, 3′, 4′
6′	122.1, CH	7.76, dd (2.0, 8.5)	2, 2′, 4′
MeO-3′	56.0, CH_3	3.99, s	3′
MeO-4′	56.0, CH_3	4.00, s	4′
MeO-5	60.2, CH_3	3.90, s	5
MeO-6	60.7, CH_3	3.95, s	6
MeO-7	56.0, CH_3	3.99, s	7

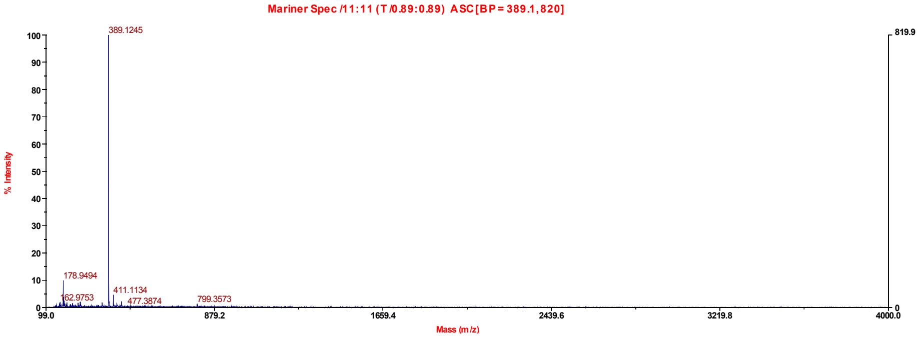

Figure S21. HDMS spectrum for CP4.

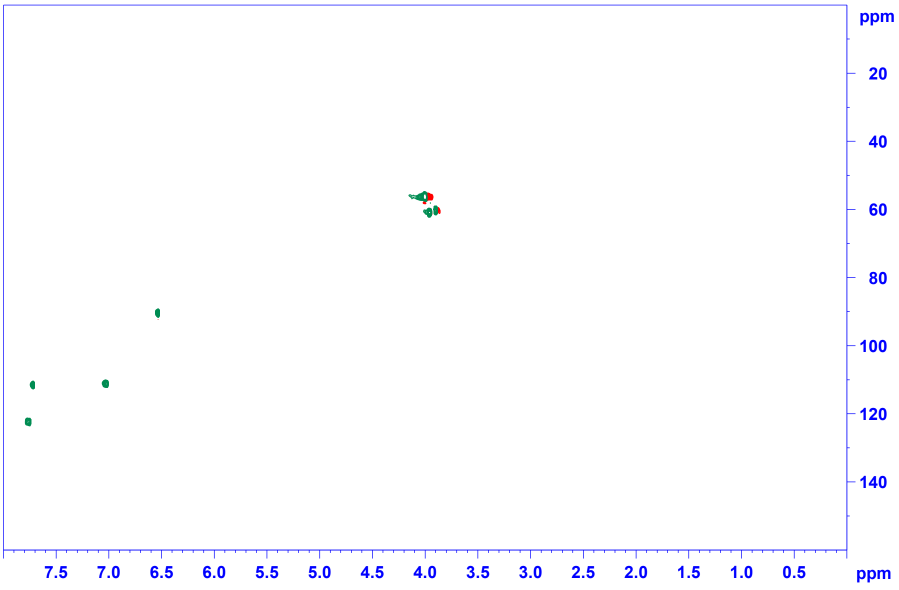

Figure S22. HSQC spectrum for CP4.

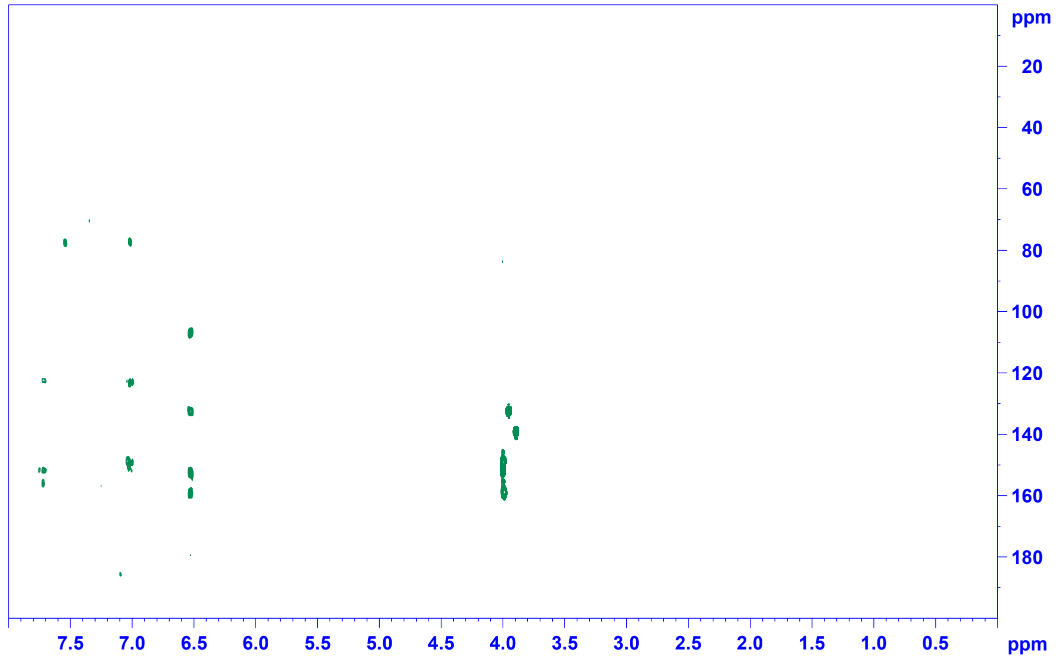

Figure S23. HMBC spectrum for CP4.

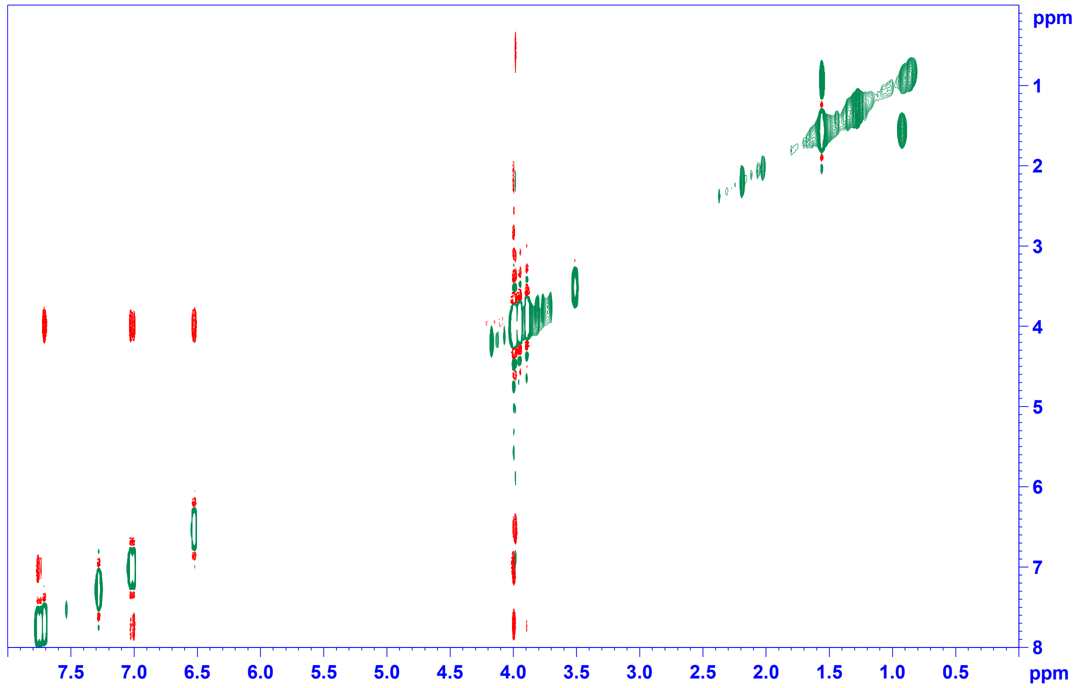

Figure S24. COSY spectrum for CP4.

5. Structure and spectral data of salvigenin (CP5)

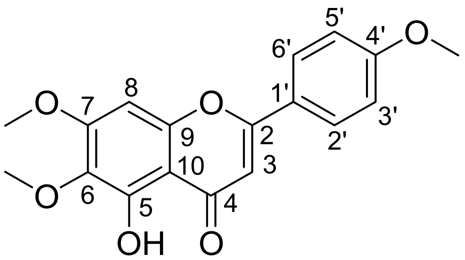

5-hydroxy-6,7-dimethoxy-2-(4-methoxyphenyl)chromen-4-one (Salvigenin) (CP5)

$M_W = 328$

MS *m*/*z* (positive mode, APCI)

HRMS *m*/*z* 329.1031 (calcd for $C_{18}H_{16}O_6^+$, 329.1025)

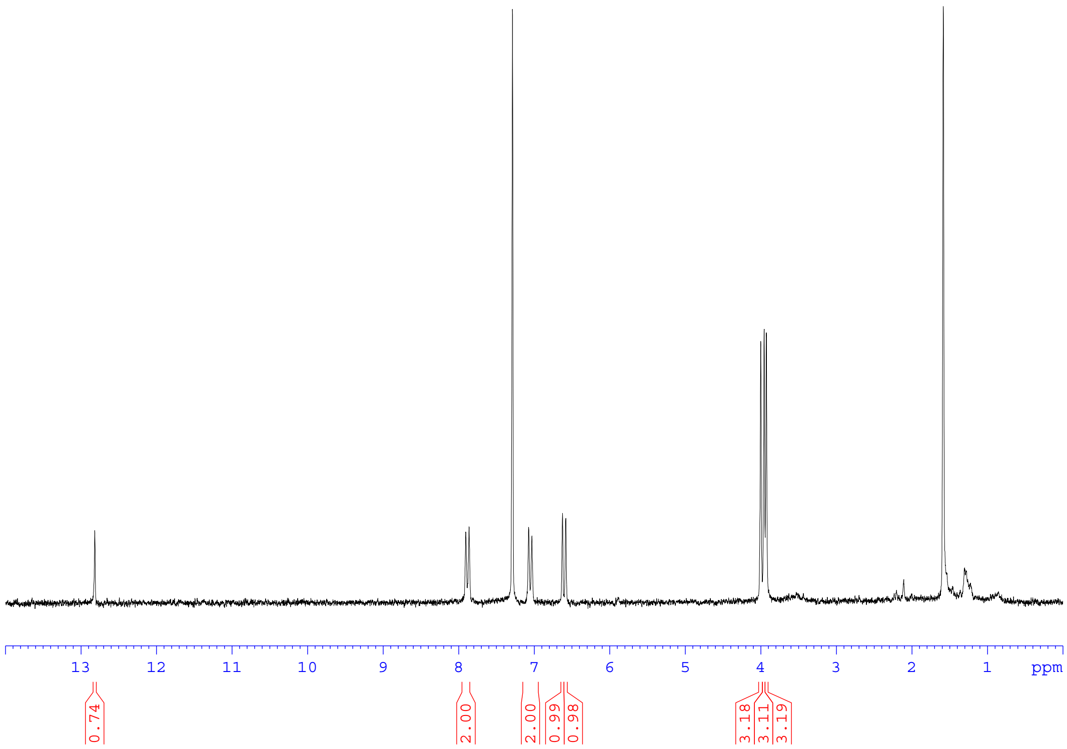

Figure S25. ^{1}H-NMR spectrum for CP5.

Table S5. Chemical shifts (δ in ppm) for CP5; Solvent: $CDCl_3$.

Position	δ_C	δ_H (*J* in Hz)	HMBC
2	163.9, C		
3	104.3, CH	6.60, s	2, 4
4	183.2, C=O		
5	153.1, C		
6	132.7, C		
7	159.0, C		
8	90.8, CH	6.56, s	4, 6, 7, 9, 10
9	152.5, C		
10	105.6, C		
1′	123.6, C		
2′	128.4, CH	7.89, d (8.2)	2, 4′, 6′
3′	114.8, CH	7.06, d (8.2)	1′, 5′
4′	163.1, C		
5′	114.8, CH	7.06, d (8.2)	1′, 3′
6′	128.4, CH	7.89, d (8.2)	2, 2′, 4′
MeO-4′	56.0, CH_3	3.90, s	4′
MeO-6	61.3, CH_3	3.93, s	6
MeO-7	56.7, CH_3	3.97, s	7

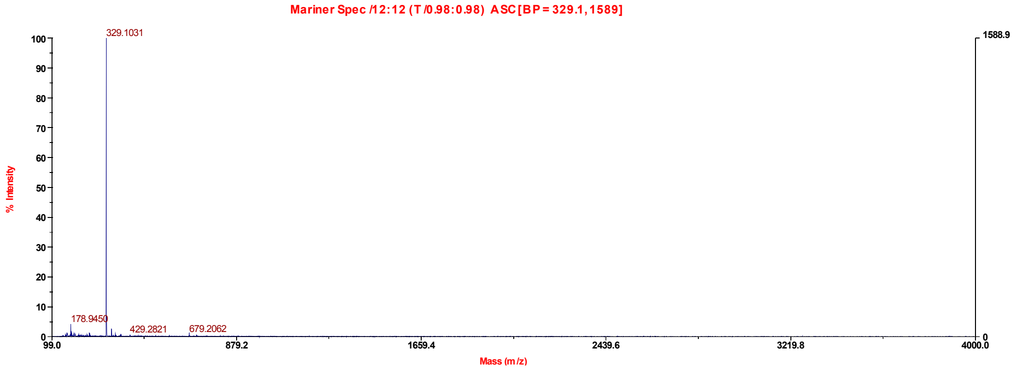

Figure S26. HRMS spectrum for CP5.

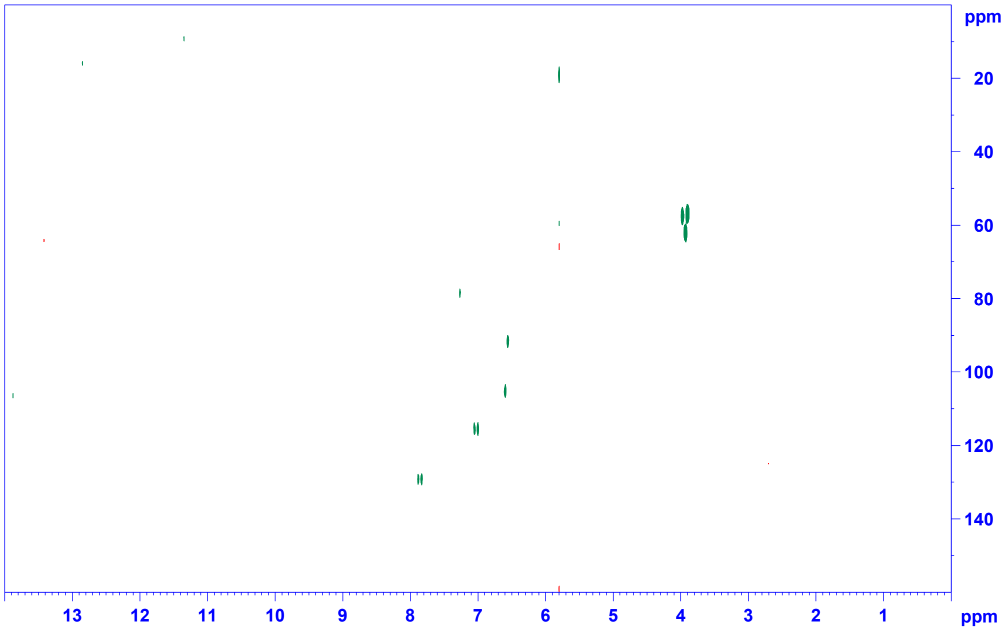

Figure S27. HSQC spectrum for CP5.

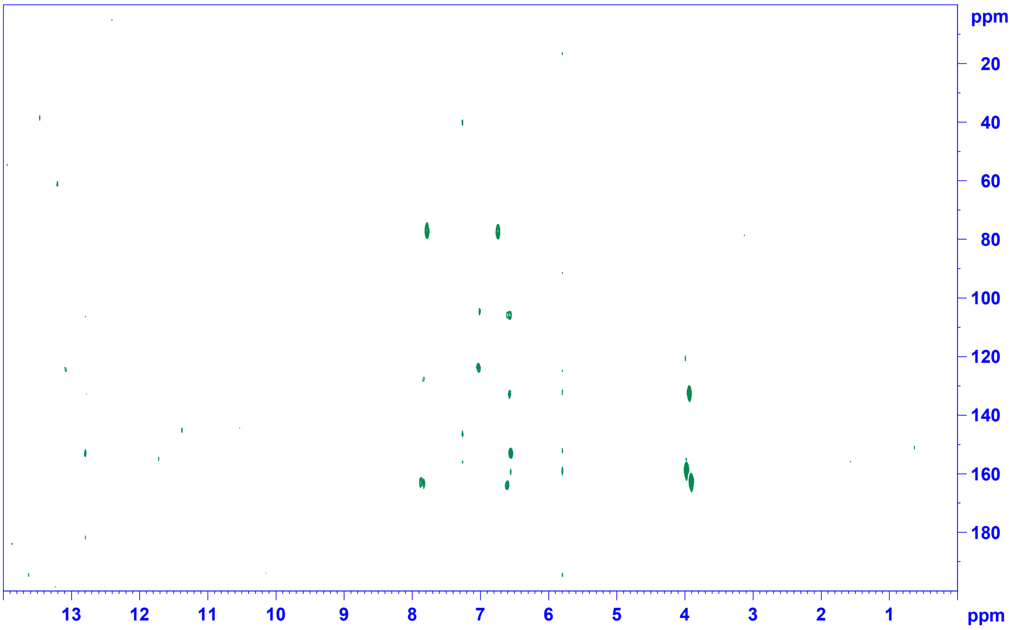

Figure S28. HMBC spectrum for CP5.

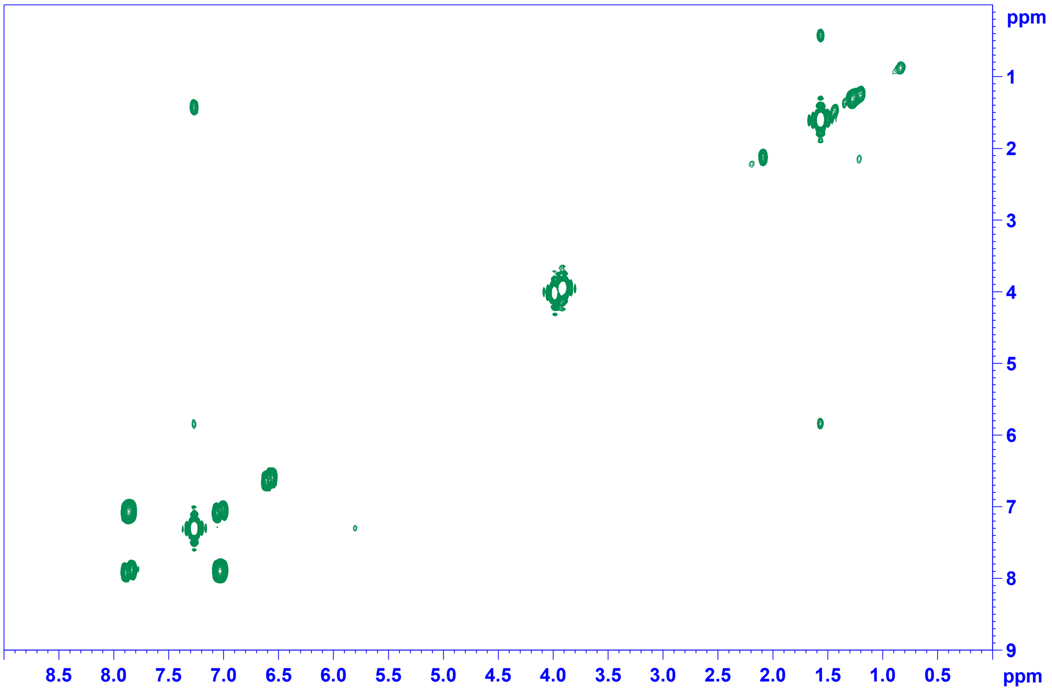

Figure S29. COSY spectrum for CP5.

Table S6. Summary of the changes in the mice weight.

Day	Healthy Group	Diabetic Group	EAF Treated Group	HAE Treated Group	CF Treated Group	WE Treated Group	Quercetin Treated Group
	Weight (g)	Weight (g)	Weight (g)	Weight (g)	Weight (g)	Weight (g)	Weight (g)
0	24.77 ± 1.47	24.87 ± 1.64	25.06 ± 2.91	25.07 ± 2.43	24.53 ± 0.49	24.66 ± 2.11	25.06 ± 2.69
4	26.52 ± 1.44	24.47 ± 2.68	25.37 ± 2.83	25.02 ± 2.43	23.43 ± 1.25	24.59 ± 1.69	25.45 ± 2.75
8	27.53 ± 1.13	24.91 ± 2.87	25.57 ± 2.60	25.43 ± 2.30	21.13 ± 2.31	24.84 ± 1.82	25.95 ± 2.93
12	28.53 ± 0.83	24.21 ± 2.18	25.67 ± 2.60	25.57 ± 2.41	18.20 ± 0.71	24.91 ± 1.63	26.08 ± 2.96
16	29.70 ± 0.85	24.83 ± 2.04	25.90 ± 2.71	25.47 ± 2.30	-	25.41 ± 1.84	26.83 ± 3.06
20	30.83 ± 0.64	24.86 ± 1.75	26.37 ± 2.57	25.93 ± 2.61	-	25.86 ± 1.52	27.88 ± 3.09

Each value is mean ± SD for six mice in each group. The empty cells in the table are because the mice in the CF treated group didn't survive past day 15.

Table S7. Tentative identification of phenolic compounds in *A. wilhelmsii*.

Compound with Literature Reference	t_R(min)	HPLC DAD λ Max (nm)	Molecular formula	$[M-H]^-$ (m/z)	MS^2(m/z)
Isoschaftoside [*,a,c] [1–3]	6.6	270, 338	$C_{26}H_{28}O_{14}$	563	545, 503, 473, 443, 383, 353
Schaftoside [*,a,a] [1–3]	6.7	270, 340	$C_{26}H_{28}O_{14}$	563	545, 503, 473, 443, 383, 353
Vicenin-2 [a,b] [1–3]	7.0	271, 335	$C_{27}H_{30}O_{15}$	593	503, 473, 383, 353, 297
Vicenin-3 [a,c] [1,4]	7.3	270, 336	$C_{22}H_{22}O_{10}$	563	443, 383, 353
Caffeic acid [*,a,b,c] [5]	7.7	221, 325	$C_9H_8O_4$	179	135
Isoorientin [a, b, c] [6,7]	8.7	270, 348	$C_{21}H_{20}O_{11}$	447	429, 357, 327
Isovitexin [*,a,b,c] [6,7]	8.8	271, 334	$C_{21}H_{20}O_{10}$	431	311, 283
Swertisin [b,c] [8–10]	9.0	270, 344	$C_{22}H_{22}O_{10}$	445	325
Ferulic acid [*,b,c,d] [5]	10.2	218, 312	$C_{10}H_{10}O_4$	193	178, 149

* This compound was compared with a library standard that was prepared using the same HPLC column and method. [a] This compound appears in the selected segment of the WE chromatogram; [b] This compound appears in the selected segment of the EAF chromatogram; [c] This compound appears in the selected segment of the HAE chromatogram; [d] This compound appears in the selected segment of the CF chromatogram. 1β,10β-epoxydesacetoxymatricarin (CP1), Leucodin (CP2), 5-demethylsinensetin (CP3), 2-(3,4-dimethoxy-phenyl)-3-hydroxy-5,6,7-trimethoxy-chromen-4-one (CP4) and Salvigenin (CP5) were isolated and identified individually.

References

1. Lin, Y.; Kong, L. Studies on the chemical constituents of *Desmodium styracifolium* (Osbeck) Merr. *Asian. J. Tradit. Med*. **2006**, *1*, 34–36.
2. Valant, K.; Besson, E.; Chopin, J. C-Glycosylflavones from the genus *Achill*. *Phytochem*. **1978**, *17*, 2136–2137.
3. Negri, G.; Santi, D.D.; Tabach, R. Chemical composition of hydroethanolic extracts from *Siparuna guianensis*, medicinal plant used as anxiolytics in Amazon region. *Rev. Bras. Farmacogn*. **2012**, *22*, 1024–1034.
4. Benayad, Z.; Gómez-Cordovés, C.; Es-Safi, N.E. Characterization of flavonoid glycosides from Fenugreek (*Trigonella foenum-graecum*) crude seeds by HPLC–DAD–ESI/MS analysis. *Int. J. Mol. Sci.* **2014**, *15*, 20668–20685.
5. Abu-Reidah, I.M.; Ali-Shtayeh, M.S.; Jamous, R.M.; Arráez-Román, D.; Segura-Carretero, A. Comprehensive metabolite profiling of *Arum palaestinum* (Araceae) leaves by using liquid chromatography-tandem mass spectrometry. *Food Res. Int*. **2015**, *70*, 74–86.
6. Mohamed, T.K.; Kamal, A.M.; Nassar M.I.;Ahmed, M.A.E.; Haggag, M.G.; Ezzat, H.A.M. Phenolic contents of *Gleditsia triacanthos* leaves and evaluation of its analgesic, anti-inflammatory, hepatoprotective and antimicrobial activities. *Life Sci. J*. **2013**, *10*, 3445–3466.
7. Zhou, C.; Luo, J.G.; Kong, L.Y. Quality evaluation of *Desmodium styracifolium* using high-performance liquid chromatography with photodiode array detection and electrospray ionisation tandem mass spectrometry. *Phytochem. Anal*. **2012**, *23*, 240–247.
8. McCormick, S.; Mabry, T.J. O-and *C*-glycosylflavones from *Passiflora biflora*. *Phytochemistry* **1983**, *22*, 798–799.
9. Dadheech, N.; Soni, S.; Srivastava, A.; Dadheech, S.; Gupta, S.; Gopurappilly, R.; Gupta, S. A small molecule swertisin from *Enicostemma littorale* differentiates NIH3T3 cells into Islet-Like clusters and restores normoglycemia upon transplantation in diabetic Balb/c mice. *Evid. Based Complement. Altern. Med*. ***2013***, 1–20.
10. Wallace, J.W.; Morris, G. *C*-Glycosylflavones in *Gnetum gnemon*. *Phytochemistry* **1978**, *17*, 1809–1810.
